# Stable Sn-Based Hybrid Perovskite-Related Structures with Tunable Color Coordinates via Organic Cations in Low-Temperature Synthesis

**DOI:** 10.1021/acsenergylett.3c00791

**Published:** 2023-05-16

**Authors:** Aarya Prabhakaran, Balaji Dhanabalan, Iryna Andrusenko, Andrea Pianetti, Simone Lauciello, Mirko Prato, Sergio Marras, Pavlo Solokha, Mauro Gemmi, Sergio Brovelli, Liberato Manna, Milena P. Arciniegas

**Affiliations:** † Center for Convergent Technologies, Istituto Italiano di Tecnologia, Via Morego 30, 16163 Genova, Italy; ‡ Dipartimento di Chimica e Chimica Industriale, Università degli Studi di Genova, Via Dodecaneso, 31, 16146 Genova, Italy; ∥ Electron Crystallography, Center for Materials Interfaces, Istituto Italiano di Tecnologia, Viale Rinaldo Piaggio 34, 56025 Pontedera, Italy; ⊥ Dipartimento di Scienza dei Materiali, Università degli Studi di Milano-Bicocca, via R. Cozzi 55, 20125 Milano, Italy

## Abstract

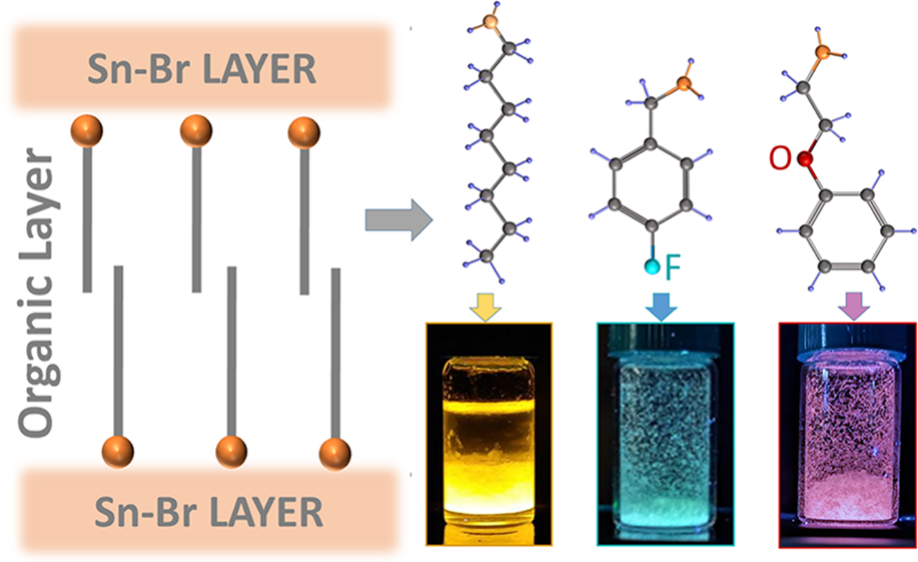

Organic–inorganic Pb-free layered perovskites are efficient broadband emitters and thus are promising materials for lighting applications. However, their synthetic protocols require a controlled atmosphere, high temperature, and long preparation time. This hinders the potential tunability of their emission through organic cations, as is instead common practice in Pb-based structures. Here, we present a set of Sn–Br layered perovskite-related structures that display different chromaticity coordinates and photoluminescence quantum yield (PLQY) up to 80%, depending on the choice of the organic monocation. We first develop a synthetic protocol that is performed under air and at 4 °C, requiring only a few steps. X-ray and 3D electron diffraction analyses show that the structures exhibit diverse octahedra connectivity (disconnected and face-sharing) and thus optical properties, while preserving the organic–inorganic layer intercalation. These results provide key insight into a previously underexplored strategy to tune the color coordinates of Pb-free layered perovskites through organic cations with complex molecular configurations.

Three-dimensional (3D) organic–inorganic metal-halide perovskites are a research hotspot in the development of materials for displays, sensors, and photovoltaics.^1,2^ In view of their simple fabrication, low cost, and tunable emission across the whole visible spectrum, these materials have driven the advancement of a new generation of solar cells and light-emitting diodes with impressive performance values.^3,4^ Although some examples have been recently reported,^5,6^ the poor structural stability of these 3D perovskites, along with the presence of Pb,^7,8^ is still limiting their real-world applications.

Conversely, low-dimensional organic–inorganic metal-halide perovskites that are formed by the intercalation of large organic cations between inorganic layers (termed layered perovskites)^9^ show higher robustness against air, heat, and moisture compared to their 3D counterparts,^10,11^ and this can be attributed to the hydrophobic character of the organic cations forming the organic layer.^12^ The intercalation of large organic cations is also responsible for their quantum and dielectric confinement, large exciton binding energy, tunable band gap, and emission from blue to cold/warm white.^13,14^ The insertion of large organic cations can generate structural distortions of the inorganic layer, breaking the conventional corner-sharing connectivity of octahedra observed in the often called 2D or quasi-2D layered perovskites,^15,16^ leading to other types of octahedral connectivity (for example, edge and face sharing or disconnected octahedra), while unusually preserving the intercalation of organic and inorganic layers,^17−20^ and thus, they can be referred to as layered perovskite-related structures.

Among the isovalent choices for Pb substitutes (e.g., Sn^2+^, Bi^2+^, Cu^2+^, Zn^2+^, Mg^2+^, and Mn^2+^)^21^ that are less toxic, Sn^2+^ is a very promising candidate, as it has electronic configuration and ionic radius similar to those of Pb^2+^, and thus, it supports analogous lattice structures.^22,23^ However, the oxidation from Sn^2+^ to the more stable Sn^4+^ upon air exposure induces the formation of SnX_4_ gas (with X representing the halide ion) in, for example, the preparation of thin films under annealing, which results in Sn^2+^ vacancies (along with X^–^ ones). This leads to materials with high defect density and poor stability under ambient conditions. Recent studies have shown how the presence of large organic cations in Sn-based organic-inorganic layered perovskites enhances the stability of the Sn-halide layers.^24−26^ Yet, their fabrication remains challenging, as it requires the stabilization of Sn^2+^ during the synthesis, and therefore, synthetic protocols developed so far rely on the use of a protected atmosphere and/or stabilizing agents such as n-trioctylphosphine,^27−29^ which however diminish the impact of using nontoxic Sn^2+^ as metal cation since trioctylphosphine itself is highly toxic.

To produce stable Sn-based low-dimensional organic–inorganic layered perovskites with less toxic stabilizing agents, Wang et al. reported an aqueous acid–based protocol, which requires heating at 80 °C and hypophosphorous acid to prevent oxidation/hydrolysis of Sn^2+^ and use octylammonium (OctA) as the organic cation.^25^ This strategy led to (OctA)_2_SnBr_4_ 2D (corner-sharing octahedra) layered perovskites exhibiting near-unity PLQY and that remained stable for over 240 days. A similar approach was recently implemented by Li et al., who showed the effect of tailoring the initial precursors and demonstrated the application of these materials as warm-white emitters.^30^Table S1 shows a compilation of reported Sn-based organic–inorganic layered perovskites (mostly 2D), and it includes synthesis conditions to highlight the energy demand and PLQY stability over time.

Although these works represent important advancements toward the synthesis of stable Pb-free layered perovskites, the color of the emission remains in the orange-yellow region of the visible spectrum, limiting their widespread use in solid-state lighting applications. In this context, changes in the molecular configuration of organic monocations have become a powerful tool to generate structural changes in Pb-based layered perovskites and thus variations in their optoelectronic properties.^31,32^ However, this approach has been underexplored in Sn-based organic–inorganic layered perovskites, and current research is limited to the use of linear primary alkylamines with different lengths in 2D layered perovskites,^25,27,29,33^ their combined use with short organic monocations to form multiple inorganic layers,^34,35^ and aromatic organic cations (without heteroatoms) in a few cases.^18,26^ The exploration of different types of organic cations as a tool for tuning the color of the emission in such low-dimensional layered materials will benefit from the development of synthetic protocols performed at low-temperatures, without the need for an inert atmosphere, and in a few steps for faster screening. These remain also critical factors to achieve low-cost Sn-based organic–inorganic layered perovskites to facilitate their integration in solid-state lighting devices.

Here we report a set of stable Sn–Br organic–inorganic layered perovskite-related structures that show different emission colors, from orange-red to bluish-green, depending on the choice of the organic cation, with only small changes in their PLQY for over a month. For this, we developed a synthetic protocol that is performed at low temperature (ca. 4 °C) and is based on a few steps to minimize the fabrication time: (i) dissolution of the initial precursors, (ii) addition of the selected amine, and (iii) cooling in an ice bath for 10 min. We initially used OctA as an organic cation and prepared platelet-like crystals with a PLQY of ca. 81% and a broad PL peak centered at 603 nm. Next, we explored a series of organic cations through this rapid synthesis strategy and found that 2-phenoxyethylammonium (POEA), which has an oxygen atom located within the aliphatic chain connected to the phenyl ring, and 4-fluorobenzylammonium (FBA), which has a fluorine atom in the para position of the phenyl ring, provide samples that display a broad emission; in the case of the POEA sample, the PL profile result is similar to that of OctA, but it is much more Stokes shifted and with a high contribution from a red component in their color coordinates, for a total PLQY value of ca. 15%. Instead, the FBA samples exhibit a broad emission band covering the region from 400 to 600 nm, with a PLQY of ca. 3%. Both samples remain stable under ambient conditions, retaining their structure and emission profile for over a month. Such changes in the color of the emission originate from modifications in the octahedra connectivity, from disconnected ones in the OctA sample to face-sharing in the POEA and FBA ones, breaking the typical corner-sharing Sn-halide octahedra connectivity observed in 2D Sn-based organic–inorganic layered perovskites, as demonstrated by 3D electron diffraction (3D ED), which also evidences the formation of aligned inorganic slabs that are intercalated by organic layers.

## Synthesis

Inspired by a synthetic protocol previously developed by Dhanabalan et al.^36^ for the synthesis of Ruddlesden–Popper A_2_PbBr_4_ layered structures (where A is a bulky organic cation), which is performed at room temperature and under ambient atmosphere, we attempted the synthesis of Sn–Br layered structures by dissolving a stoichiometric amount of SnBr_2_ in a few microliters of HBr, 1 mL of hypophosphorous acid (H_3_PO_2_) at 50 wt % in water as stabilizing agent to prevent Sn^2+^ oxidation,^25,30^ and 2 mL of the selected solvent (toluene that is immiscible with HBr and H_3_PO_2_, or acetone, which is miscible with HBr and H_3_PO_2_), as reaction medium. To this mixture, we added a stoichiometric amount of the selected amine, and we cooled the mixture in an ice bath (4 °C) (see details in Methods). Note that we chose SnBr_2_ as Sn and Br source since SnO, which is commonly used in the synthesis of Sn-based perovskites,^25^ has poor solubility at room temperature in the selected solvents. We chose octylamine as a source of the organic cation to develop and optimize the synthesis protocol. Octylamine is among the most used amines for the intercalation of Sn-halide layers^25,27,30,33^ in layered perovskite structures, and thus, it is a convenient molecule for benchmarking the optical properties of the resulting structures.

We start this discussion with the details on the synthesis conditions followed by the structural and optical characterization of the OctA sample. The choice of the reaction medium was crucial to ensure the formation of stable crystals, as is also the case in the preparation of perovskite films for highly efficient solar cells.^22,37,38^ While acetone did not promote the formation of crystals at any of the temperatures investigated (that is, room temperature, 4 and 80 °C; see Table S2), we found that toluene promotes the formation of white translucent crystals when placing the final mixture in an ice bath for 10 min to reach 4 °C. Figure S1 displays photographs of the vials taken at different steps of the synthesis when using toluene. In these experiments, we noticed the formation of a few powdery crystals in the vial soon after the addition of octylamine, but these dissolved upon shaking. Once the closed vials were placed inside an ice bath, we observed the growth of large and white crystals within minutes. Unlike other solvents such as dimethylformamide (DMF), dimethyl sulfoxide (DMSO), and *N*-methylformamide (NMF) that are fully miscible with HBr and H_3_PO_2_ and do not promote crystal formation (see synthesis conditions in Table S2), toluene forms an interface with the mixture of SnBr_2_, HBr, and H_3_PO_2_ (in water), with toluene as the top liquid layer.

To elucidate the role of toluene in the formation of crystals, we first ran the synthesis without it, following the same protocol described above. As a result, we obtained pale-yellow platelets, which emitted yellow under ultraviolet (UV) light, but their emission was quenched soon after their preparation (within 15 min), and the crystals eventually dissolved back in solution. Also, by adding toluene to this solution after crystal dissolution, we observed the reformation of crystals (Figure S2). In parallel, we also performed a set of experiments varying the solvent miscibility with HBr and H_3_PO_2_ and solvent density (see more details in the Supporting Information and Figure S2). These experiments suggest that the formation of a solvent–antisolvent interface, with the nonpolar solvent as a top layer, effectively protects/stabilizes the Sn-based crystals against ambient degradation during the synthesis.

## Structural and Optical Characteristics

**Figure 1:**
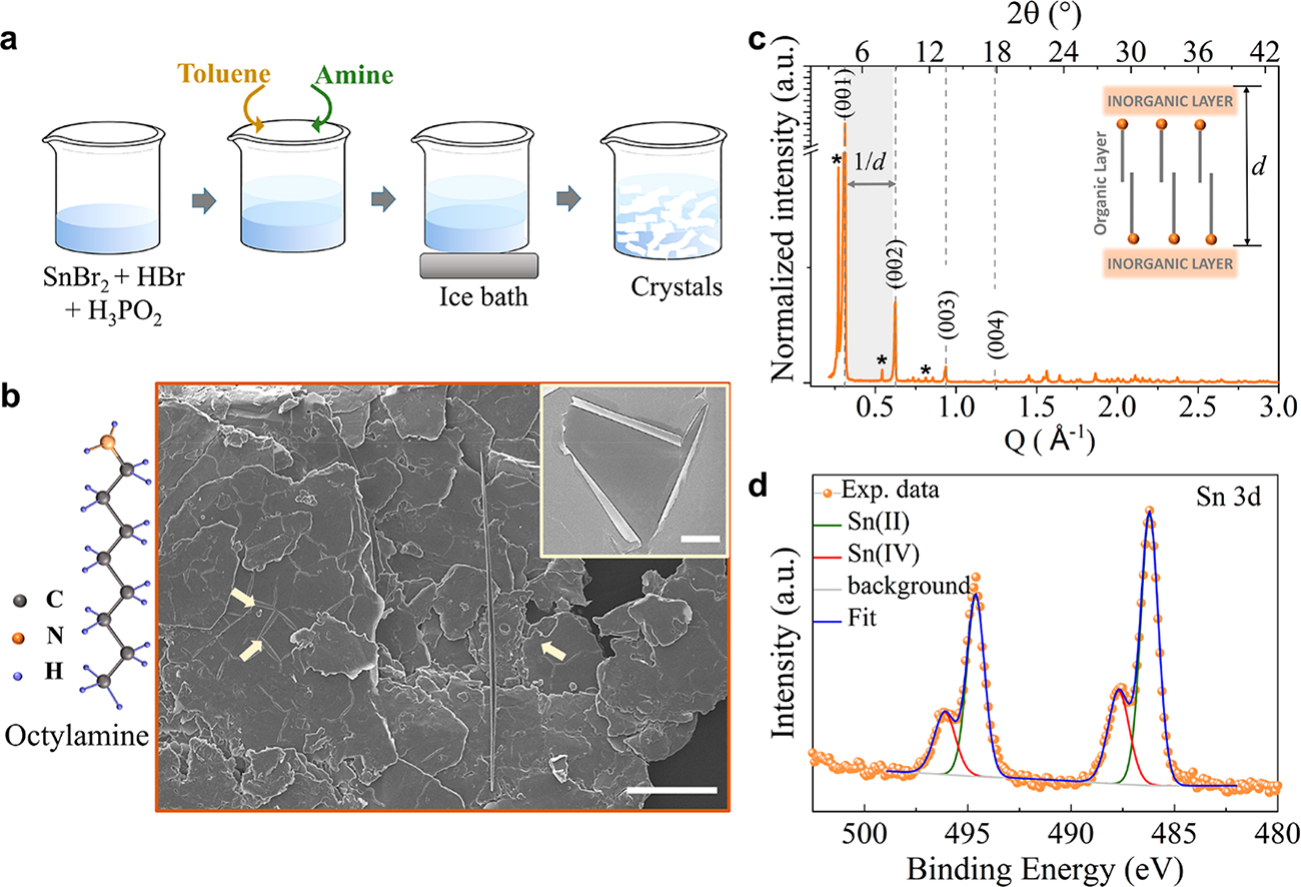
(a) Scheme illustrating the different steps of the synthesis protocol, starting from the dissolution of SnBr_2_ in HBr and H_3_PO_2_, followed by the addition of toluene and then the selected amine, to the cooling of the mixture in an ice bath. (b) SEM image of the as-synthesized OctA sample. The image shows an aggregate of platelets packed one on top of the other, parallel to the substrate. Scale bar: 100 μm. The arrows indicate the rolled edges of the platelets. The inset displays a closer view of the platelet’s edges. Scale bar: 5 μm. The sketch in panel b represents the molecular diagram of octylamine. (c) PXRD patterns collected from ground OctA sample showing periodic peaks (highlighted by dotted lines) in the (00*l*) crystallographic plane. The calculated periodicity, *d*, results in ca. 20 Å. The asterisks indicate additional periodic peaks. The embedded sketch is a representation of periodic inorganic layers separated by an organic layer. (d) XPS spectra of the Sn 3d state that were recorded from the OctA sample, evidencing the majority presence for Sn in the +2 state.

The main steps of the synthesis protocol are illustrated in Figure 1a. Figure 1b shows a representative scanning electron microscopy (SEM) image of the OctA sample, evidencing a plate-like morphology with sizes in the range from 80 to 100 μm.

The platelets stack one on top of the other and tend to roll up at the edges (inset in Figure 1b), denoting their very reduced thickness within values between 120 and 200 nm. More SEM images are presented in Figure S3. The collected powder X-ray diffraction (PXRD) patterns from both as-synthesized (Figure 1c) and ground crystals (Figure S4) show (00*l*) basal reflections that are characteristics of layered perovskite platelets laying down parallel to the substrate,^25,33^ along with reflections from other crystal orientations observed in the pattern from ground crystals. From the periodic reflections, the calculated periodicity, *d*, that is, the distance between inorganic layers, is ca. 20.15 ± 0.61 Å, which agrees with the periodicity observed from 2D layered perovskites prepared with OctA.^25,33^ We also observe three additional diffraction peaks with high intensity at low 2θ angle (at 3.8°, 7.7° and 11.5°), which remains in the PXRD pattern collected from ground samples (Figure S4). To identify these additional PXRD reflections, we compared the PXRD patterns of the fresh crystals with potential byproducts formed during the synthesis, such as octylammonium bromide, Sn_2_(H_2_PO_2_)_3_Br, and octylamine crystals (Figure S4). To elucidate if they originated from secondary products of the synthesis due to an excess of precursors, we also collected PXRD patterns from crystals prepared with different precursor concentrations (Figure S5). The results confirm that the additional diffraction peaks are not due to these impurities, and they are still present in the crystals formed by changing the content of the precursors. We noticed, however, that these periodic reflections strongly reduce their intensity after grinding the crystals for PXRD analysis (Figure S4), which indicates that they are likely related to stacks of platelets with the larger facet parallel to the substrate, as we observed from the SEM images in Figure S3. From the ground crystals (Figure S4), we observe only traces of SnO_2_ and SnBr_4_ giving reflections with very low intensity at 32.66° and 36.39°, and 28.19° and 28.51°, respectively. Note that we attempted the collection of crystallographic data by selecting large single platelets for single-crystal XRD. However, the small thickness of the structures strongly limited the sample preparation, and therefore, we performed a crystallographic analysis through 3D ED, as we will discuss later.

To investigate the composition of the crystals, we used energy-dispersive spectroscopy (EDS) in SEM (Figure S6 and Table S3), which revealed a Sn:Br ratio of 1:5.7, which is higher than the 1:4 ratio for 2D (OctA)_2_SnBr_4_ layered perovskites, suggesting a different octahedra connectivity. EDS analysis also indicated trace amounts (<2 atomic %) of phosphorus that stem from the hypophosphorous acid that is used as a reducing agent in the synthesis.

The chemical composition, including the ratio between N from OctA molecules and Br, was further assessed by high-resolution X-ray photoelectron spectroscopy (XPS; Table S4). The Sn:N and N:Br ratios of the OctA sample corresponded to 1:4.5 and 1:1.5, which additionally evidence that the Sn–Br connectivity differs from the corner-sharing one observed in 2D layered perovskites, while preserving the intercalation of organic–inorganic layers, as indicated by the PXRD analysis. We also investigated by XPS the oxidation state of the sample (Figure 1d). We identify the Sn 3d_5/2_ peaks at binding energies of 486.2 ± 0.2 and 487.7 ± 0.2 eV, which are assigned to Sn^2+^ and Sn^4+^, respectively, indicating that both oxidation states are present in the sample. However, the quantification of the corresponding peak area shows that the majority of Sn is in the form of Sn^2+^ (>70%), while the remaining percentage corresponds to Sn^4+^ which could originate from surface oxidation or be due to the presence of SnBr_4_ as an impurity in the Sn precursor, which can reach even 10% in weight.^39^ Surprisingly, the Sn^4+^ content assessed from the XPS analysis from samples in their as-synthesized condition, prepared in open air, is relatively close to the values reported for samples prepared and characterized under an inert atmosphere,^40−43^ with contribution up to 20%, as determined by the same technique and after surface etching to remove the oxidation layer. Note that we did not apply post-treatments to prevent Sn oxidation or remove the oxide layer.

To elucidate the octahedral connectivity and further confirm the intercalation of organic–inorganic layers, we performed 3D ED on all the investigated samples. This was necessary as other approaches were unsuccessful: (i) The crystals were unsuitable for single-crystal XRD measurements even when their lateral dimensions were larger than the critical size of 1 μm. (ii) Structural solution from PXRD performed on the ground platelets) failed due to the strong preferential orientation of the platelets and the large contribution from the inorganic fraction (with heavy atoms), which masked the organic fraction scattering contribution in PXRD. 3D ED instead can be efficiently used for the structure analysis of such thin crystals, and it is a reliable method for the structure determination of single submicrometric crystals. This was demonstrated in recent works on organic–inorganic 2D layered perovskites^44−46^ and metal- and covalent-organic frameworks.^47^ To avoid potential damage to the sample during the acquisition time, the experiments were performed under extremely low illumination conditions and using a nanobeam probe about 150 nm in diameter (see details in Methods).

**Figure 2:**
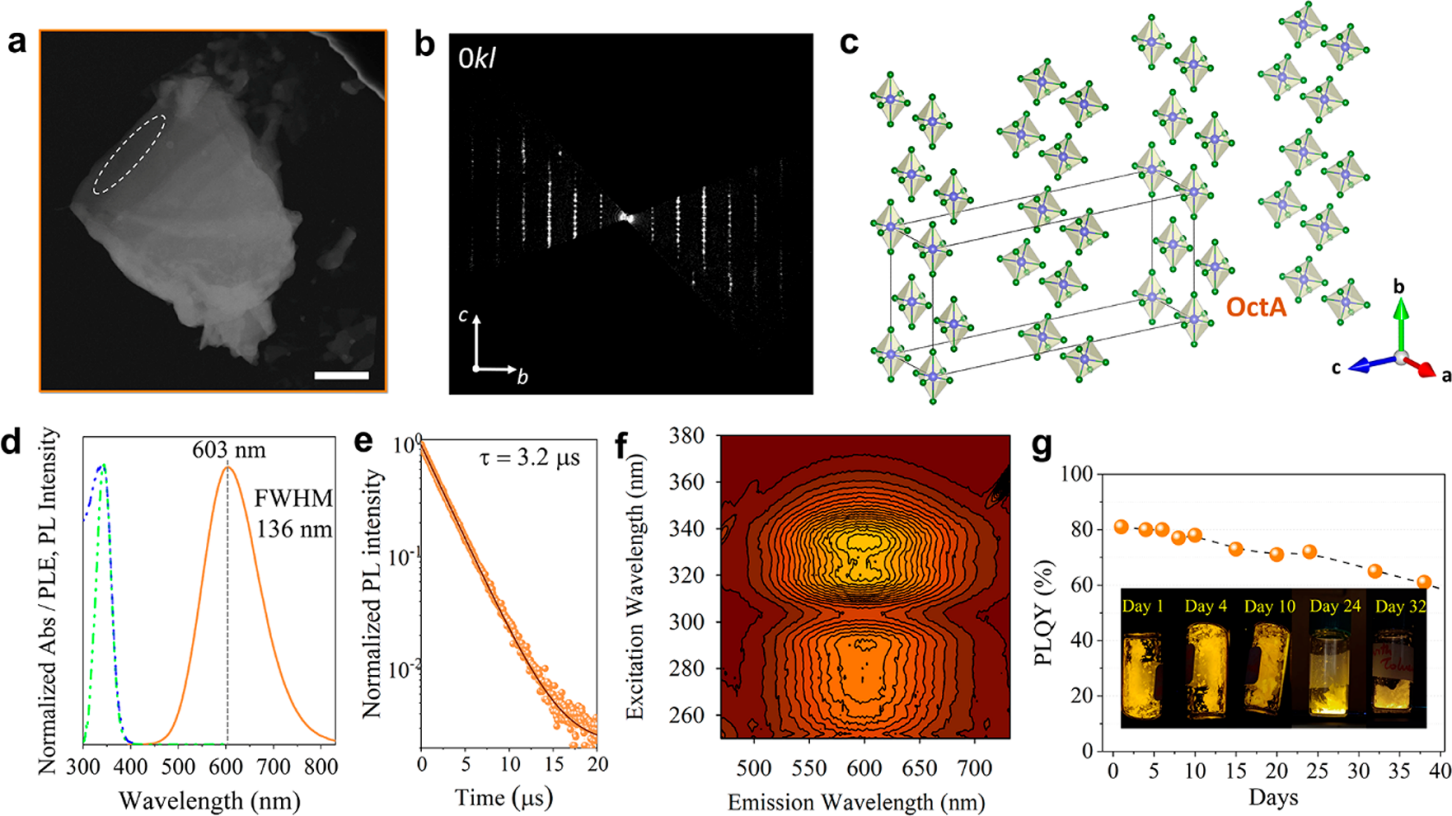
(a) HAADF-STEM image of a typical platelet of the OctA sample used for 3D ED data collection (region framed in white). Scale bar: 1 μm. (b) Exemplary planar 0*kl* cut of the 3D ED reconstructions. More planar cuts are reported in Figure S7. (c) Crystal structure of inorganic layers in the OctA sample. Sn atoms are in violet and Br atoms are in green. (d) Representative absorbance (in blue), PLE (in green), and emission (in orange) spectra. The vertical dotted line indicates the PL peak maximum. (e) Time dependence of the PL decay collected from the platelets. (f) 3D PL/PLE contour plot. (g) PLQY values as a function of time from samples stored in a closed vial under air for 35 days evidencing the ambient stability of the platelets. The embedded photographs show a representative sample under UV light at different times of storage under ambient air.

We recorded 3D ED data from several single-crystal fragments with sizes less than 1 μm (Figure 2a). Although the acquisition of 3D ED data from organic-containing samples can be challenging due to potential structural instability issues attributed to the weak forces that connect the organic and inorganic layers,^48,49^ the samples remained stable under the electron beam exposure and high TEM vacuum during the time of analysis, showing the same structural motif over time. All data sets collected from different crystals were consistent with the same *C*-centered monoclinic unit cell, with approximate cell parameters *a* = 10.1 Å, *b* = 17.8 Å, *c* = 41.2 Å, β = 91.9° (Table 1). Moreover, reciprocal space sections of 3D ED data (Figures 2b and S7) revealed no additional extinction condition, pointing convincingly to the extinction symbol *C*1–1 which is compatible with the space groups *C*2 (5), *Cm* (8) and *C*2/*m* (12). We performed a partial structure solution by standard direct methods (SDMs) and using the most complete 3D ED data set. This resulted in the automatic localization of disconnected [SnBr_6_] octahedra (Figures 2c and S8). However, organic cations were not detected automatically, and thus, we tried to determine their positions by simulated annealing (SA). Recently, such a combination of SDMs and SA has been successfully applied to 3D ED data for the structure determination of organic–inorganic layered perovskite.^46^

**Table 1:** Crystallographic Information Extracted from the 3D ED Data Collected from the Different Samples^a^

	OctA	POEA	FBA
Crystallographic Information			
Space group	*C*2*/m*	*P*1	*P*1
*Z*	8	1	1
*a* (Å)	10.1	6.5	4.5
*b* (Å)	17.8	8.9	6.1
*c* (Å)	41.2	16.4	15.5
α (deg)	90	91.9	96.4
β (deg)	91.9	94.2	100.9
γ (deg)	90	90.5	94.3
Volume (Å^3^)	7402.4	946.2	414.3
*Ab Initio* Structure Determination by *SIR2014*			
Tilt range (deg)	81	100	105
Data resolution (Å)	1.1	1.1	1.1
Sampled reflections (No.)	5448	1687	789
Independent reflections (No.)	2000	876	406
Independent reflection coverage (%)	65	60	63
Global thermal factor *U*_iso_ (Å^2^)	0.11418	0.05608	0.07412
*R*_int_ (%)	26.17	14.45	16.99
*R*_SIR_ (%)	26.92	19.37	18.94

a The table also reports the ab initio structure determinations performed via *SIR2014*.

Unfortunately, SA did not converge to any chemically sound model due to the very large cell volume of the OctA sample. Nevertheless, the best crystallographic model containing only inorganic components was obtained in space group *C*2/*m* (12). More details about structure determination are reported in Table 1. In the final structure model for the OctA sample (Figure 2c), two independent Sn–Br inorganic layers of isolated [SnBr_6_] octahedra parallel to the *a*–*b* plane are located at *z* = 0 and 0.5 in agreement with the configuration of organic–inorganic layered perovskites with disconnected octahedra.^18^ Additional views of the crystal structure are provided in Figure S8. The distance between layers of disconnected octahedra results in ca. 20 Å, matching well the periodicity obtained from the sequence of strong periodic peaks observed in the PXRD pattern collected from as-synthesized and ground crystals (Figures 1c and S3).

Next, we discuss the optical properties of the crystals that were evaluated by UV–vis absorption and PL (Figure 2d). The absorbance profile (in blue) shows a strong peak in the region from 320 to 360 nm (3.87–3.44 eV) that is characteristic of direct band gap organic–inorganic low dimensional perovskites,^50,51^ and it is centered at ca. 344 nm (3.63 eV). The emission (in orange) exhibits the distinctive broad profile reported for similar low-dimensional structures,^30,51,52^ centered in this case at ca. 603 nm (2.06 eV) with a full width at half-maximum (fwhm) of around ca. 136 nm (ca. 0.39 eV).

The absorbance and emission profile do not overlap and show a large Stokes shift of ca. 260 nm (ca. 1.56 eV) that is well in agreement with existing literature on structures produced through more complex routes.^18,30,52^

To get a better insight into the emission characteristics, we performed PL excitation (PLE) spectroscopy analysis of the sample at the maxima of the broadband emission (ca. 603 nm) and plotted the result in Figure 2d. The PLE profile shows a single peak structure that matches well with the observed absorbance peak at ca. 344 nm, indicating that this electronic state is contributing to the sample emission. Moreover, time-resolved PL decay collected at the maxima of the broadband emission and fitted using a single exponential function (Figure 2e) shows that the samples have a long decay lifetime of ca. 3.2 μs. A representative 3D PL/PLE contour plot of the sample is displayed in Figure 2f. Together, the large Stokes shift, broadband emission, and long PL decay observed from the samples prepared with octylamine indicate that self-trapped excitons (STE) are mediating the recombination mechanism from these disconnected octahedra forming the inorganic layers.^10,18,30,50^

The as-synthesized crystals show a PLQY of ca. 81% ± 5% obtained from three independent measurements from three different batches of samples. This relatively high PLQY value denotes that the simple protocol implemented for their synthesis does not compromise their optoelectronic properties. Moreover, the sample’s PLQY remains stable for the initial 10 days with a small reduction to values around 70% after 25 days and with a drop to 65% after 35 days (Figure 2g) when storing them (from two different synthesis batches) in a closed vial at ambient conditions. In addition, we collected PXRD patterns from the samples after 7, 13, and 42 days, and after 7 months to track potential air-induced degradation of the structures. There is a slight decrease in the intensity of the XRD peaks in the patterns collected at 13 and 42 days (see details in Figure S9). After 7 months, the reflections observed in the fresh samples disappear and the sample is not emitting anymore under UV illumination. We also observe the appearance of PXRD peaks after 42 days that increase in intensity with time from the fresh sample, which are attributed to the air-driven decomposition of Sn into SnO_2_, as well as the formation of SnBr_4_,^53,54^ and potentially other Sn-based complexes from reflections observed at low angles. These results show that the samples undergo a slow degradation process starting at around 2 weeks of being preserved under ambient conditions, as seen in the small changes observed in the PLQY values.

## Investigating Organic Cations with Different Molecular Structures

To translate the knowledge acquired from the Pb-based systems to Sn-based ones and tune the color of their emission,^32^ we investigated a set of amines with different molecular structures through the protocol developed for octylamine (Tables S5 and S6). This set included amines containing heteroatoms based on the following criteria: the presence of heteroatoms in the organic cations may induce dipole moment/charge separation in the structure,^55^ which could in turn impart different distortions to the Sn–Br octahedra and therefore originate STE relaxation pathways for PL tunability.^16^ We found that both 2-phenoxyethylamine and 4-fluorobenzylamine induce the formation of crystals through the same synthesis protocol with a relatively longer crystal formation time (ca. 30 min) compared to that of octylamine.

**Figure 3:**
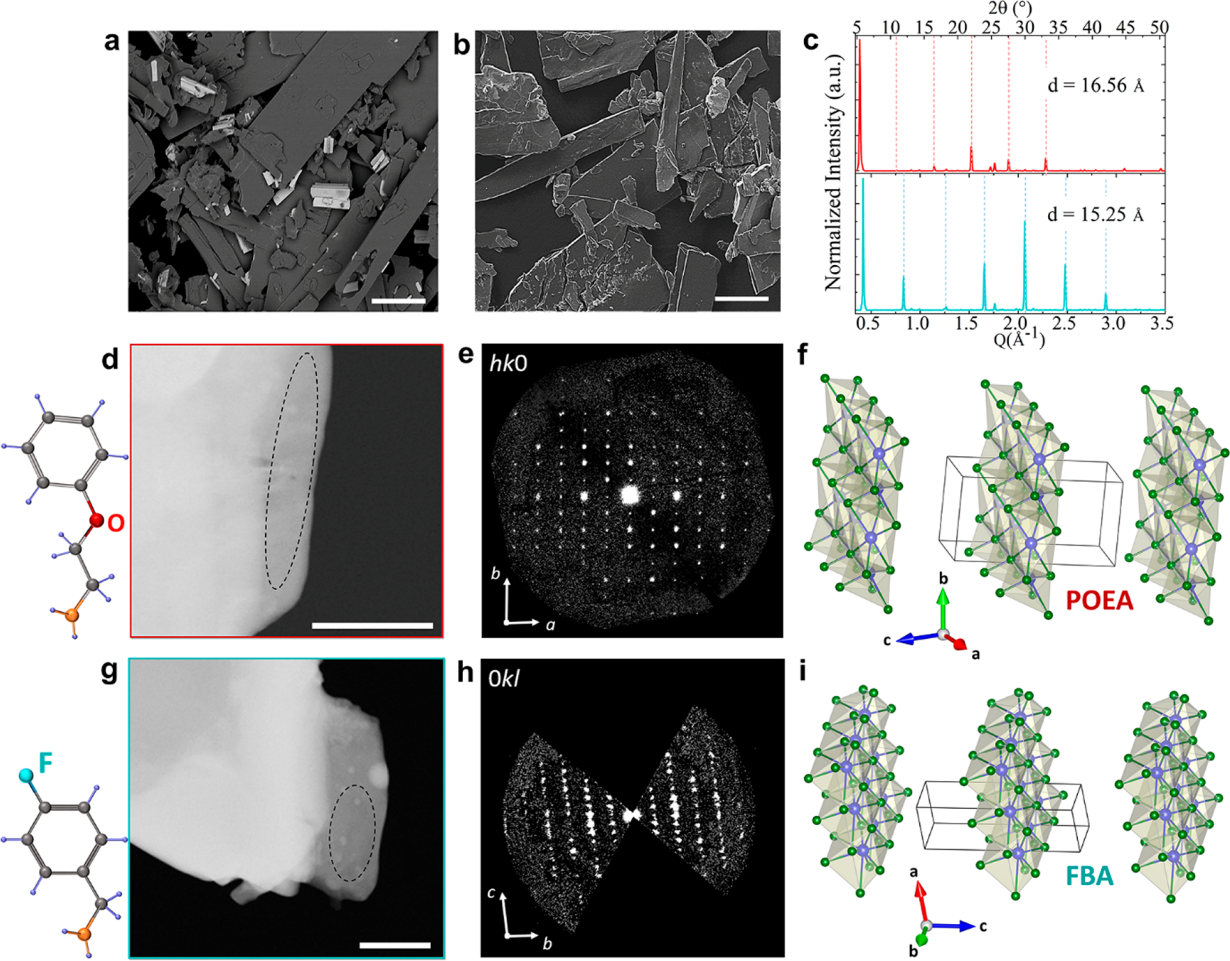
(a and b) Representative SEM images of the POEA (a) and FBA (b) samples. Scale bars: 100 μm. (c) PXRD patterns collected from ground POEA and FBA samples showing (00*l*) periodic reflections (highlighted by parallel dotted lines). (d and g) HAADF-STEM images of typical platelets of POEA (d) and FBA (g) samples. 3D ED data were collected from regions framed in black. (e and h) Exemplary planar cuts of the 3D ED reconstructions for the corresponding samples. More planar cuts are reported in Figures S11 and S12. (f and i) Crystal structures of inorganic layers in the POEA (f) and FBA (i) samples. Sn atoms are in violet and Br atoms are in green.

The crystals are characterized by an elongated platelet-like morphology with their long side length varying from 50 to 350 μm crystals (see SEM images in Figure 3a,b). The collected XRD patterns from these samples evidence periodic reflections (even from ground platelets as shown in Figure 3c), with *d* values of 16.56 ± 0.048 Å for POEA structures and 15.25 ± 0.44 Å for FBA ones. These inter-distances are comparable with reported values for 2D layered perovskites formed with POEA (16.42 Å)^56^ and FBA (15.25 Å)^57,58^ as organic cations in Pb- and Cu-based structures, respectively. The EDS analysis reported in Figures S10 and S11 and Tables S7 and S8 indicates an excess of Br in the POEA (Sn:Br ratio of 1:6), while the Sn:Br ratio in the FBA samples is 1:4.

Analysis of the oxidation states of the samples was performed via XPS. With these organic cations, we observe peaks in the XPS spectra (Figure S12 and Table S9) that are shifted toward higher binding energy compared to those from the OctA sample: Sn 3d_3/2_ at 486.8 ± 0.2 and 487.9 ± 0.2 eV for the POEA sample and at 486.6 ± 0.2 and 487.5 ± 0.2 eV for FBA sample, which are assigned to Sn^2+^ and Sn^4+^, respectively. Such a shift in binding energy can be attributed to potential distortions of the Sn–Br octahedra layer.^27^ We also observe a higher Sn^4+^ content in the samples prepared with these organic cations, around 55–75%, indicating that they are less efficient at protecting a distorted inorganic layer; therefore, post-treatments such as the addition of a graphene layer^59^ or additive engineering^24^ may help to prevent further Sn oxidation.

Further crystallographic details were obtained via 3D ED. All data sets confirm the presence of parallel inorganic layers separated by a distance of 16.4 Å for POEA and 15.5 Å FBA samples, in agreement with the periodicities obtained from PXRD. In both cases, 3D ED data (Figure 3e,h and Figures S13 and S15) delivered a triclinic unit cell with parameters reported in Table 1. Note that *a* and *b* in-plane lattice parameters of FBA sample are more contracted compared to the POEA sample, which points toward changes in the accommodation of each amine at the anchor side. This in turn strongly influences their emission properties, as we will discuss below.

For both samples, Sn–Br inorganic layers were recognized and placed parallel to the *a–b* plane (Figure 3f,i). The examination of the main planes of 3D ED reciprocal space reconstruction (Figures S13 and S15) confirms the triclinic nature of both samples. The most accurate crystallographic model of the inorganic layers was obtained by SDMs in space group *P*1 (1). Note that in contrast with the samples prepared with octylamine, these structures have face-sharing octahedra, resembling the connectivity reported for perovskitoids^60,61^ (see closer views of the structures in Figures S14 and S16).

**Figure 4:**
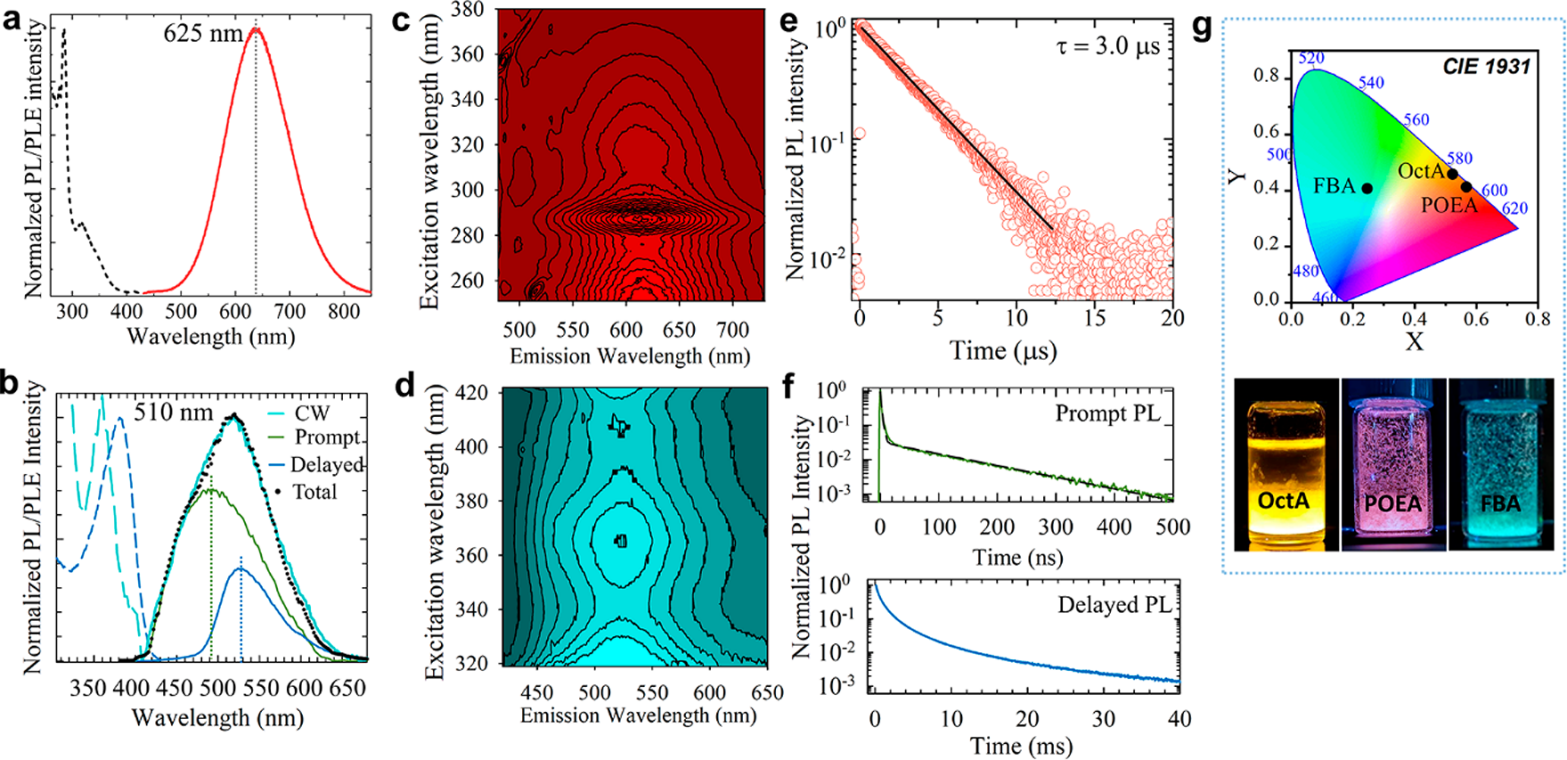
(a and b) Representative PL (solid and dotted lines) and PLE (dashed lines) spectra collected from POEA (a) and FBA crystals (b). In panel b, the color of the PL curve refers to the time scale: in black, PL measured under continuous wave (CW) excitation; in green, time-gated PL (*t* = 0–100 μs) contribution recorded synchronously to excitation; and in blue, delayed PL contribution for *t* > 300 μs. The black dots are the sum of the prompt and delayed PL contributions matching well the CW PL spectrum. (c and d) The corresponding 3D-PL/PLE contour plots of POEA (b) and FBA (c). (e) PL decay traces collected at the PL maximum of POEA. The black line is the corresponding fitting to a single exponential function. (f) Time decay curves of the prompt and delayed PL from FBA crystals collected at the wavelength indicated by the vertical dotted lines in panel b. The black line in the top panel is the corresponding fitting to a bi-exponential function. (g) CIE chromaticity diagram displaying the corresponding coordinates obtained from the different layered structures. The photographs show the vials containing the emitting crystals.

We then investigated the optical properties of POEA and FBA crystals to elucidate potential changes induced by their crystallographic structures, that is, the type of organic cations and octahedra connectivity, compared to OctA crystals. Figure 4a,b reports representative emission spectra and PLE of the POEA (Figure 4a) and FBA crystals (Figure 4b). The contour plots of the PLE/PL for both samples are displayed in Figure 4c,d.

The POEA sample exhibits emission properties similar to OctA crystals, both in terms of PL profile (with a slight red-shift to 625 nm and a fwhm of ca. 136 nm) and single exponential decay dynamics (τ = 3.0 μs, Figure 4e). However, the PLE spectrum is markedly different, with an intense narrow peak at 285 nm and a much weaker PLE band at 350 nm, which matches well the PLE spectrum of the OctaA structure. The FBA crystals, in turn, exhibit substantially weaker PL peaked in the green region (at 510 nm, Figure 4b), indicating that the emissive state is different from the other structures. Consistently, the PL decay is overall significantly faster, with a single exponential tail with a characteristic lifetime of τ_1_ = 130 ns and an initial fast portion (τ_2_ = 3 ns). While the FBA crystals display a very low PLQY of ca. 3%, POEA ones reach 15% of PLQY. Based on the low PLQY value of FBA and the weight ratio of the slower versus faster contributions (I_1_/I_2_ = 0.02), we ascribe the latter to nonradiative quenching by trap sites and the first to radiative exciton decay. Interestingly, and in agreement with the presence of nonradiative traps, the PL spectrum of the FBA sample acquired with a continuous wave (CW) laser shows a significant contribution by delayed fluorescence, possibly originating from the slow release of trapped carriers. This is evidenced by time-gated PL/PLE measurements (collected after ∼100 μs from the excitation pulse) reported as a blue line in Figure 4b. It shows a markedly asymmetrical PL peak at ∼540 nm and multiexponential decay dynamics with an effective lifetime of 800 μs (measured as the time after which the intensity has dropped by a factor *e*) (see the bottom panel in Figure 4f).

The observed changes in PL profiles from POEA and FBA impact the Commission Internationale de l’Eclairage (CIE) coordinates of the crystals, from (0.52, 0.46) of the OctA crystals to (0.25, 0.41) when using FBA as organic cations to build the structures (Figure 4g). Although OctA and POEA share similar optical features, their color coordinates evidence a high contribution from the red channel in the POEA crystals. Photographs of the samples under UV light are displayed in Figure 4g. Moreover, both samples retained their crystallographic structure (Figure S17) and emission for ca. 7 months stored under ambient conditions (Figure S18).

In summary, we fabricated a new set of Sn–Br layered perovskite-related structures that show different emission colors, from yellowish-orange to orange-red, to blue-green by incorporating three different organic cation parts of the amine family, from conventional alkylammonium ones to cyclic cations with heteroatoms. We developed a facile synthesis protocol, which is performed at low temperatures and under ambient conditions, that allows the rapid screening of different organic cations in the synthesis of Sn-based hybrid structures. The observed changes in the color chromaticity of the synthesized structures are attributed to the different Sn–Br octahedra connectivity, from fully disconnected to face-sharing, depending on the choice of organic monocations. The tuning of the color of the emission through organic cations in stable Pb-free perovskite-related structures with good optical properties via a simple synthesis protocol may grant access to their application in solid lighting applications. Our work also highlights the relevance of structural engineering to achieve desirable optoelectronic properties of layered hybrid materials.

## Methods

### Materials

Octylamine (99%), 2-phenoxyethylamine (98%), 4-fluorobenzylamine (97%), hydrobromic acid (48% aq.), hypophosphorous acid (50% aq.), SnBr_2_, DMF, DMSO, NMF, acetone, methanol, ethanol, and toluene were purchased from Sigma-Aldrich and employed without further purification.

### Synthesis

SnBr_2_ (72.3 mg, 0.26 mmol) was dissolved in aqueous HBr 40% by weight (140 μL, 1.2 mmol) in a 4 mL glass vial. Then, H_3_PO_2_ 50% by weight (1 mL) was added to the mixture, resulting in a clear transparent solution after shaking, followed by the addition of toluene (2 mL). The solution was strongly shaken using a vortex for a few seconds. Once the toluene interface was visible, the selected amine (1.2 mmol – 198 μL of octylamine; 157 μL of 2-phenoxyethylamine; and 137.13 μL of 4-fluorobenzylamine) was rapidly injected. The resulting mixture was shaken for 1 min, and immediately after, the vial was placed in an ice bath for 10 min. The crystals were dried in a Buchner funnel using a vacuum pump by first depositing them in filter paper. The dried crystals were transferred to a 4 mL glass vial, and toluene (2 mL) was added. The crystals in toluene were centrifuged at 5000 rpm for 5 min, and the supernatant was discarded. This step was repeated three times.

### Preparation of Ammonium Bromide Salts

The ammonium bromide salts were prepared by using a modified literature procedure.^62^ Briefly, HBr (1.03 mL, 0.02 mol) was added dropwise to a solution prepared with the selected amine (0.02 mol) in 4 mL of ethanol under magnetic stirring at 4 °C for 3 h. The resulting white powders were collected and washed with diethyl ether in a Buchner funnel using a vacuum pump.

### Structural Characterization

Powder X-ray diffraction (PXRD) patterns were collected from 5° to 60° with a step size of 0.013° by using a parallel beam geometry with Cu Kα radiation (λ = 1.541874 Å) in an Empyrean diffractometer (Malvern PANalytical, Westborough, MA), working at 45 kV and 40 mA and equipped with a PIXcel 3D 2 × 2 area detector. For the measurements, well-dried samples placed on a zero-diffraction silicon substrate were used. Measurements were also performed on ground crystals to reduce the intensity of the strong (00*l*) basal reflections from the platelets and to acquire signals from other crystallographic planes. PXRD patterns from 3° to 60° were collected in Bragg-Brentano geometry, using a third-generation Empyrean diffractometer equipped with automated prefix iCore-dCore optical modules for the incident and diffracted beam paths.

The morphology of the samples was analyzed by using a field emission scanning electron microscopy (FE-SEM) JEOL JSM-7500 FA (Jeol, Tokyo, Japan), operating at 25 kV acceleration voltage and considering backscattering electrons for enhancing differences in chemical composition. Energy-dispersive spectroscopy (EDS, Oxford instrument, X-Max, 80 mm^2^ SDD detector) was used to distinguish the presence and the ratio of Sn, P, and Br in the samples.

The chemical composition and oxidation state of the main elements present in the samples were investigated via X-ray photoelectron spectroscopy (XPS). XPS analyses were carried out with a Kratos Axis Ultra^DLD^ spectrometer using a monochromatic Al Kα source (20 mA, 15 kV). High-resolution XPS spectra were collected at a pass energy of 20 eV, with an analysis area of 300 × 700 μm, preserving short acquisition times (minutes for each spectrum) to avoid potential damage of the samples under X-ray irradiation. Spectra were analyzed using CasaXPS software (version 2.3.24). The fitting of the Sn 3d spectra was obtained considering that, due to spin–orbit coupling, each Sn chemical state corresponds to a pair of peaks (Sn 3d_5/2_ and Sn 3d_3/2_) having the same full width at half-maximum, a spin–orbit splitting of 8.41 eV, and an area ratio of 3:2 between the 5/2 and 3/2 components. The position of the Sn 3d doublet was conveniently identified by the position of the 3d_5/2_ component. Specimens for XPS were prepared by pressing dried samples onto conductive copper tape.

High-angle annular dark-field scanning transmission electron microscopy (HAADF-STEM) imaging and three-dimensional electron diffraction (3D ED) were carried out with a transmission electron microscopy (TEM) Zeiss Libra 120 equipped with a thermionic LaB_6_ source (120 kV). 3D ED was performed in STEM mode after defocusing the beam to have a parallel illumination on the sample.^63^ ED patterns were collected in Köhler parallel illumination with a beam size diameter of about 150 nm, obtained using a 5 μm C2 condenser aperture. Data were recorded by a single-electron ASI Timepix detector,^64^ which delivers virtually background-free diffraction patterns and allows working with a very low electron dose (around 0.05 el Å^–2^ s^–1^) to minimize the beam damage to the sample. The as-synthesized samples were gently crushed and loaded directly on a carbon-coated Cu TEM grid without any solvent or sonication. 3D ED acquisitions were performed at room temperature, in both continuous and stepwise modes.^65^ No evidence of beam damage, such as fading of high-resolution reflections or the appearance of amorphous rings, was ever observed even in stepwise mode (which requires a higher total exposure time per experiment). This was likely due to the extremely low dose rate of the illumination setup and the relative beam stability of the materials. Further details are reported in the Supporting Information. Crystallographic views were extracted from Vesta software.^66^

### Optical Characterization

Samples for the optical studies were prepared by placing the crystals between two clean fused quartz glass microscopic slides. 3D PL and PLE were collected in time-tagged mode with an Agilent Cary Eclipse spectrophotometer. Prompt/fast emissions (below 100 μs total decay time) were measured in fluorescence mode, and delayed/slow emissions (from 100 μs to 500 ms total decay time) were measured in phosphorescence mode.

Photoluminescence spectra were collected under continuous xenon lamp excitation coupled with a double additive grating scanning monochromator Gemini 180. The sample’s emission was collected and focalized with a lens doublet to entrance slits of a Triax 180 monochromator coupled with a Peltier-cooled CCD camera.

For time-resolved fluorescent measurements, the samples were excited at PLE maximum using a Hyperion femtosecond laser from Ultrafast coupled with an Apollo optical parametric amplifier. The sample’s emissions were collected using an Oriel Instrument Cornerstone 1/4 m monochromator coupled with a Hamamatsu UV–Vis photomultiplier and a time-correlated single-photon counting unit.

The PLQY values were obtained from each sample by exciting them at the same wavelength as for collecting the PL using a calibrated integrating sphere with step increments of 1 nm and an integration time of 0.2 s per data point for five repeated measurements. Light absorption due to scattering inside the sphere was taken into account for the PLQY calculation by collecting three different spectra: (1) directly exciting the sample in the sphere, (2) indirectly exciting the sample in the sphere, and (3) without the sample in the sphere.

The optical absorbance spectrum of the OctA sample was collected by using a Varian Cary 5000 UV–vis–NIR spectrophotometer equipped with an external diffuse reflectance accessory and operating in the absorption geometry by employing the integrating sphere.

## Supporting Information Available

A detailed table containing the existing literature, highlighting the synthesis procedure and properties of Sn-based hybrid perovskites; table containing the trial experiments to optimize the synthesis protocol; additional SEM images; further details on 3D ED; table summarizing trials performed with different organic cations; EDS data; additional PXRD patterns (PDF)
