# Supplementary material for: Stable Sn-Based Hybrid Perovskite-Related Structures with Tunable Color Coordinates via Organic Cations in Low-Temperature Synthesis
Source: ACS Energy Lett. 2023 May 16;8(6):2630–40. doi: 10.1021/acsenergylett.3c00791 (PMC10262684; doi:10.1021/acsenergylett.3c00791)
Supplement: Supplementary file 1 — nz3c00791_si_001.pdf [file nz3c00791_si_001.pdf]

Supporting Information for

Stable Sn-based Hybrid Perovskite-related Structures with Tunable Color Coordinates via Organic Cations in Low-Temperature Synthesis

*Aarya Prabhakaran,[†§] Balaji Dhanabalan,[†˥] Iryna Andrusenko,[¥] Andrea Pianetti,[£] Simone Lauciello,[†] Mirko Prato,[†] Sergio Marras,[†] Pavlo Solokha,[§] Mauro Gemmi,[¥] Sergio Brovelli,[£] Liberato Manna[†] and Milena P. Arciniegas[†]**

[†]Center for Convergent Technologies, Istituto Italiano di Tecnologia, Via Morego 30, 16163, Genova, Italy.

[§]Dipartimento di Chimica e Chimica Industriale, Università degli Studi di Genova, Via Dodecaneso, 31, 16146, Genova, Italy

[¥]Electron Crystallography, Center for Materials Interfaces, Istituto Italiano di Tecnologia, Viale Rinaldo Piaggio 34, 56025, Pontedera, Italy

[£]Dipartimento di Scienza dei Materiali, Università degli Studi di Milano-Bicocca, via R. Cozzi 55, 20125 Milano, Italy.

**Correspondence should be addressed to milena.arciniegas@iit.it.*

Table S1. Comparison of key features of recently reported Sn-based halide layered perovskites with different octahedra connectivity, from the traditional corner-sharing ones (2D) to disconnected octahedra, including the structures synthesized in the present work. Synthesis conditions highlight the energy demand and the stabilizing agent if used. The column labeled 'stability' refers to the preservation of the PLQY value over time; -- indicates unreported data; RT: room temperature.

Structure	Organic cation	Synthesis conditions	PL peak nm	PLQY %	Stability days	Ref.
2D $(C_6H_{13}NH_3)_2SnBr_4$	Hexylamine	Hot Injection, N_2 atm, 180°C	618	35	--	[1]
2D $(C_8H_{17}NH_3)_2SnBr_4$	Octylamine	Aqueous acid based, 80°C, H_3PO_2	600	95	240	[2]
		Hot Injection, N_2 atm, 180°C	610	82	--	[1]
		Ambient air, 100°C, 0°C, H_3PO_2	612	54	--	[3]
2D $(C_{12}H_{25}NH_3)_2SnBr_4$	Dodecylamine	Hot Injection, N_2 atm, 180°C	603	60	--	[1]
		Ambient air, 100°C, 0°C, H_3PO_2	616	2	--	[3]
2D $(C_{18}H_{35}NH_3)_2SnBr_4$	Oleylamine	Hot Injection, N_2 atm, 180°C	623	50	30	[1]
		Hot Injection, N_2 atm, 180°C, tri-n-octylphosphine	620	88<br>68 in films	10	[4]
		Ambient air, 100°C, 0°C, H_3PO_2	628	60	--	[3]
2D $(C_{18}H_{37}NH_2)_2SnBr_4$	Octadecylamine	Ambient air, 100°C, 0°C, H_3PO_2	617	2	--	[3]

**Face-sharing Sn-Br intercalated with POEA**	**2-Phenoxyethylamine**	**Ambient air, 4°C, H_3PO_2**	**625**	**15**	**30**	**This work**
**Face-sharing Sn-Br intercalated with FBA**	**4-Fluorobenzylamine**		**510**	**3**	**30**	**This work**
2D $(C_8H_{10}NH_2)_2SnBr_4$	Phenylethylamine	Slow cooling, N_2 atm, 100°C, H_3PO_2	470	<0.1	--	[5]
2D $(C_8H_{10}NH_2)_2SnI_4$		Acid precipitation, N_2 atm, 100°C, H_3PO_2	625, 680	--	--	[6]
2D $(C_4H_{14}NH_2)_2SnI_4$	Butylamine	Acid precipitation, N_2 atm, 80°C, H_3PO_2	620, 680	--	2	[6]
2D $(C_4H_{14}NH_2)_2SnI_{4+X}$		Aqueous acid based, 100°C, H_3PO_2	632	<1	--	[7]
			578	89	PLQY 55% after 15 days	
2D $(C_6H_{10}NH_2)_2SnI_4$	Hexylamine	Acid precipitation, N_2 atm, 90°C, H_3PO_2	620, 665	--	2	[6]
		Aqueous acid based, 100°C, H_3PO_2	633	<1	--	[7]
2D $(C_6H_{10}NH_2)_2SnI_{4+X}$			598	99	PLQY 81% after 15 days	
2D $(C_8H_{16}NH_2)_2SnI_4$	Octylamine	Acid precipitation, N_2 atm, 140°C, H_3PO_2	620, 670	--	--	[6]
2D $(C_8H_{16}NH_2)_2SnI_{4+X}$		Aqueous acid based, 100°C, H_3PO_2	640	<1	--	[7]
			617	92	PLQY 86% after 15 days	

Disconnected octahedra $(PEA)_4SnBr_6$	Phenylethylamine	Solvent diffusion, N_2 atm, RT	566	89.5	--	[5]
**Disconnected Sn-Br octahedra intercalated with OctA**	**Octylamine**	**Ambient air, 4°C**	**603**	**80**	**30**	**This work**

Table S2. Synthesis conditions tested through the protocol described in the main text. RT: room temperature.

Solvent – volume, mL	H_3PO_2, mL	Temperature, °C	Observations
Acetone – 2	0	RT	No crystal formation
	1	RT	
	1	80	
	1	4	
	0	4	
DMF – 2	0	RT	No crystal formation
	1	4	No crystal formation
DMSO – 2	0	RT	No crystal formation
	1	4	No crystal formation
THF – 2	0	RT	No crystal formation
	1	4	No crystal formation
NMF – 2	0	RT	No crystal formation
	1	4	No crystal formation
Toluene – 2	0	RT	Small amount yellow precipitate - no emission
	0.5	4	Yellowish powder more sticky - emitting yellow under UV - not stable emission quenched in few min
	1	RT	No formation – a thin layer of white powder formed at the interface after a few min - not emitting
	1	4	White crystals emitting bright yellow under UV lamp
P-Xylene - 2	1	4	White crystals emitting bright yellow under UV lamp

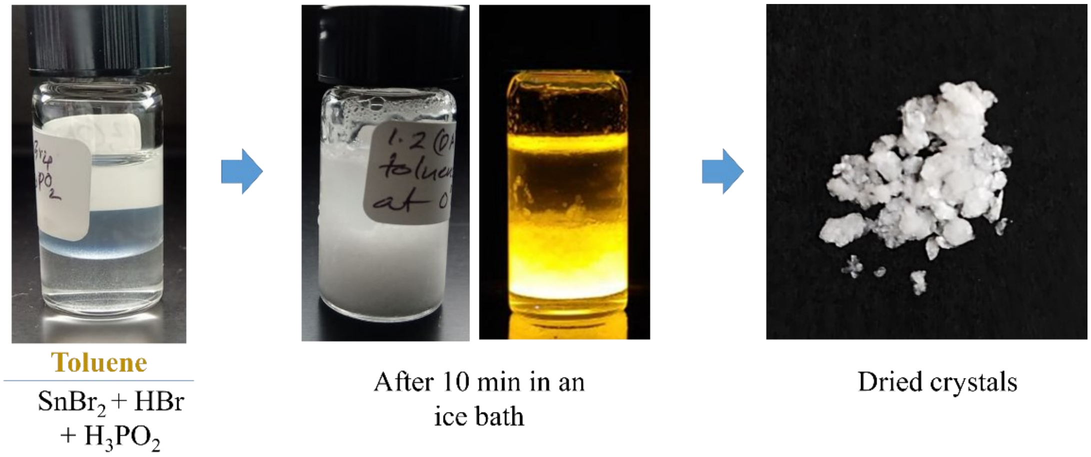

Figure S1. Photographs of the vials taken at different steps of synthesis when using octylamine. From left to right: Vial containing $SnBr_2$ dissolved in HBr + H_3PO_3 + toluene before adding the amine. The interface between the toluene (top layer) is clearly visible; (center panel) crystals formed in solution after adding octylamine and keeping the vial for 10 minutes in an ice bath. Photos were taken under normal (right) and UV light (left); and finally, a photo of the washed and dried crystals.

Role of the solvent in the crystal formation when using octylamine

We replaced toluene (0.867 g/mL density) with other non-polar solvents such as p-xylene (0.875 g/mL density), diethyl ether (0.713 g/mL density) and ethyl acetate (0.902 g/mL density) in the synthetic protocol described in the main text. Diethyl ether or ethyl acetate did not promote crystal formation, while crystals were observed when using p-xylene (Figure S2). This denotes that both the density of the solvents and the relative difference in solvent miscibility with the polar solvents present in the mixture (HBr and water from the stabilizing agent) determine the effectiveness of the selected solvent to promote the reaction.

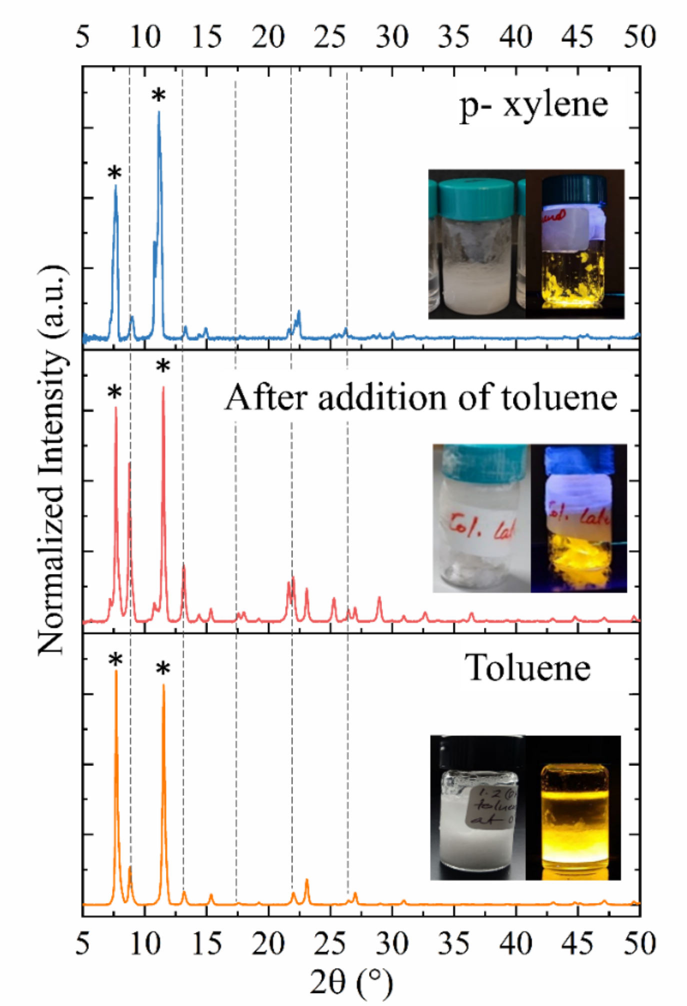

Figure S2. PXRD patterns collected from the as-synthesized OctA sample (in orange), the crystals obtained by adding toluene after the injection of the amine (without toluene in the initial mixture) (in red), and crystals prepared with p-xylene instead of toluene in the synthesis (in blue). The photographs show the vials under normal (left) and UV (right) light. The asterisks in the panels highlight the additional reflections that are attributed to stacks made of platelets parallel to the substrate, and the dotted lines represent the characteristic periodic peaks from periodic inorganic layers.

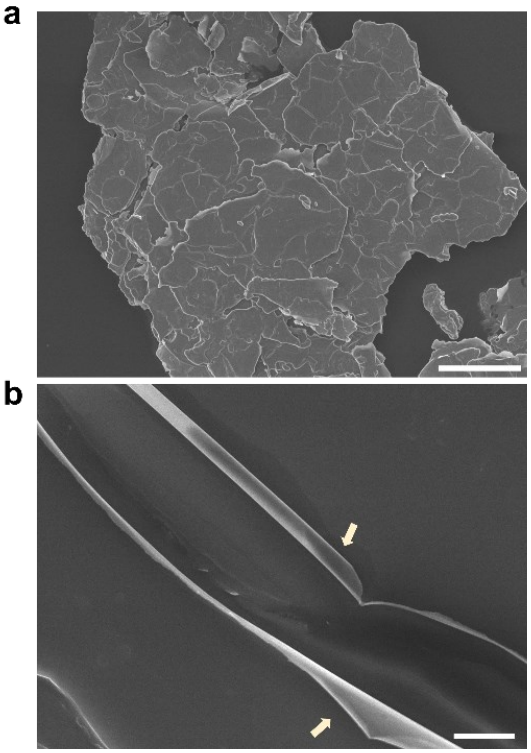

Figure S3. Additional SEM images of the OctA sample showing a group of platelets stacking one on the top of the other (a) and a closer view of rolled-up edges of platelets indicated by arrows (b). Scale bars: 100 µm (a) and 2 µm (b).

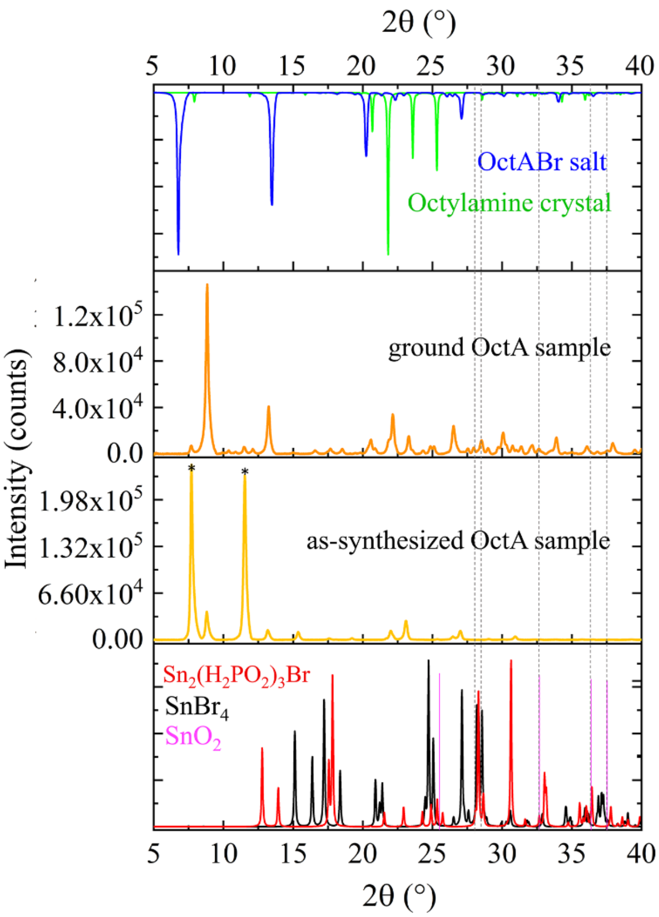

Figure S4. PXRD patterns collected from the as-synthesized OctA sample (in yellow) and compared to the PXRD patterns collected from ground OctA crystals, as well as other potential compounds such as octylammonium bromide salt, octylamine crystals (COD 7214425), $Sn_2(H_2PO_2)_3Br$ structures,[8] and products of Sn decomposition under ambient air, such as SnO_2 (ICSD 98-018-1109) and $SnBr_4$ (ICSD-98-002-6033). The additional reflections observed in the as-synthesized sample (highlighted by asterisks in the yellow pattern) remain in the ground sample, and they do not correspond with any of the potential byproducts. The preparation of the salt is described in the Methods section.

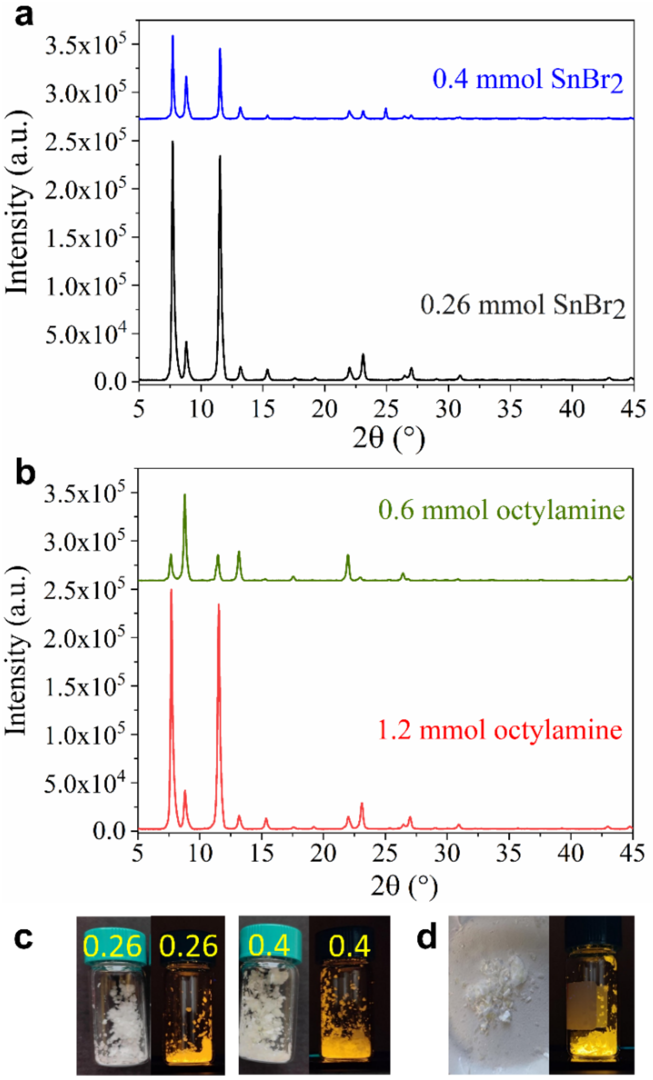

Figure S5. **a-b,** PXRD patterns collected from samples obtained by changing the amount of $SnBr_2$ in the synthesis at a fixed concentration of octylamine of 1.2 mmol (a) and by changing the amine concentration in the synthesis at a fixed $SnBr_2$ concentration of 0.26 mmol (b). Compared to the crystals produced through the synthesis conditions reported in the main document, the structures produced by these synthesis conditions show less intense diffraction peaks in the PXRD patterns, indicating a relatively lower crystallinity. **c-d** Corresponding photographs taken under normal (left) and UV light (right) of the samples prepared with different $SnBr_2$ (c) and amine concentration (d).

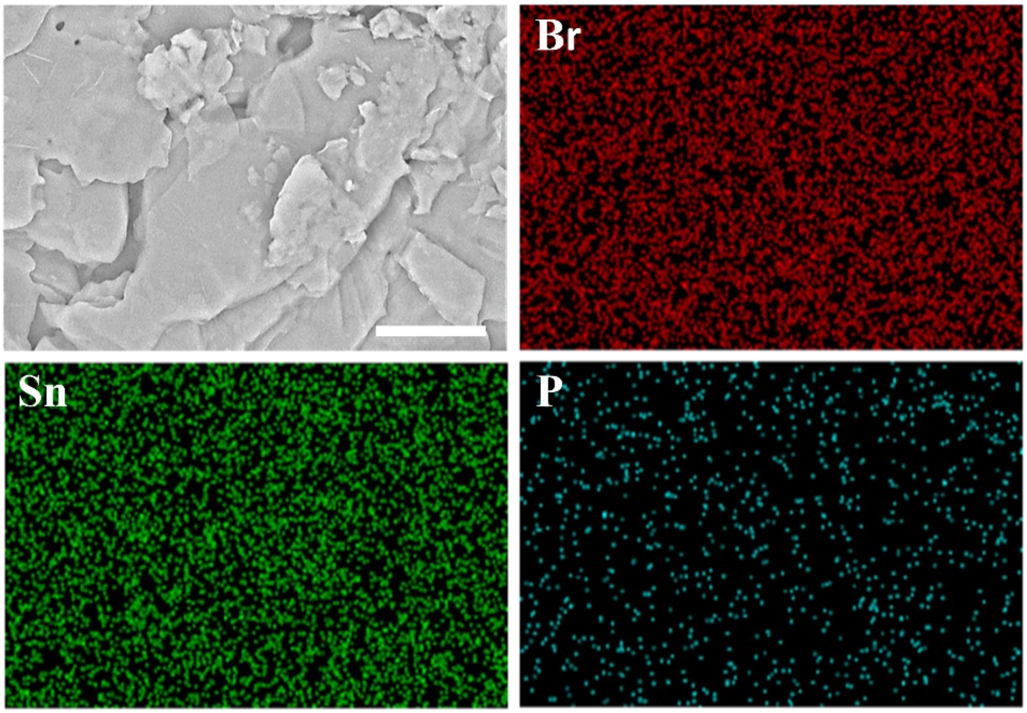

Figure S6. (top-left) SEM image of a representative area showing a group of OctA platelets and the corresponding EDS-SEM elemental mapping performed for Br and Sn. Traces of P are also detected in the sample (bottom-right panel) that are attributed to residual H_3PO_2 used in the synthesis. Scale bar: 50 µm.

Table S3. Elemental mapping analysis for Sn, Br, and P obtained via EDS-SEM performed on the OctA sample. According to this quantification, the resulting Sn:Br ratio in atomic % is 1:5.73.

Map Sum Spectrum	Line Type	Wt%	Wt% Sigma	Atomic %
Sn	L series	20.43	0.50	14.57
Br	K series	78.89	0.53	83.56
P	K series	0.68	0.23	1.87
Total		100.00		100.00

Table S4. Relative atomic percentage for O, C, N, Sn, and Br, obtained via XPS performed on the OctA sample.

Element	Relative atomic %
**O**	2.6
**C**	79.7
**N**	6.4
**Sn**	1.4
**Br**	9.9

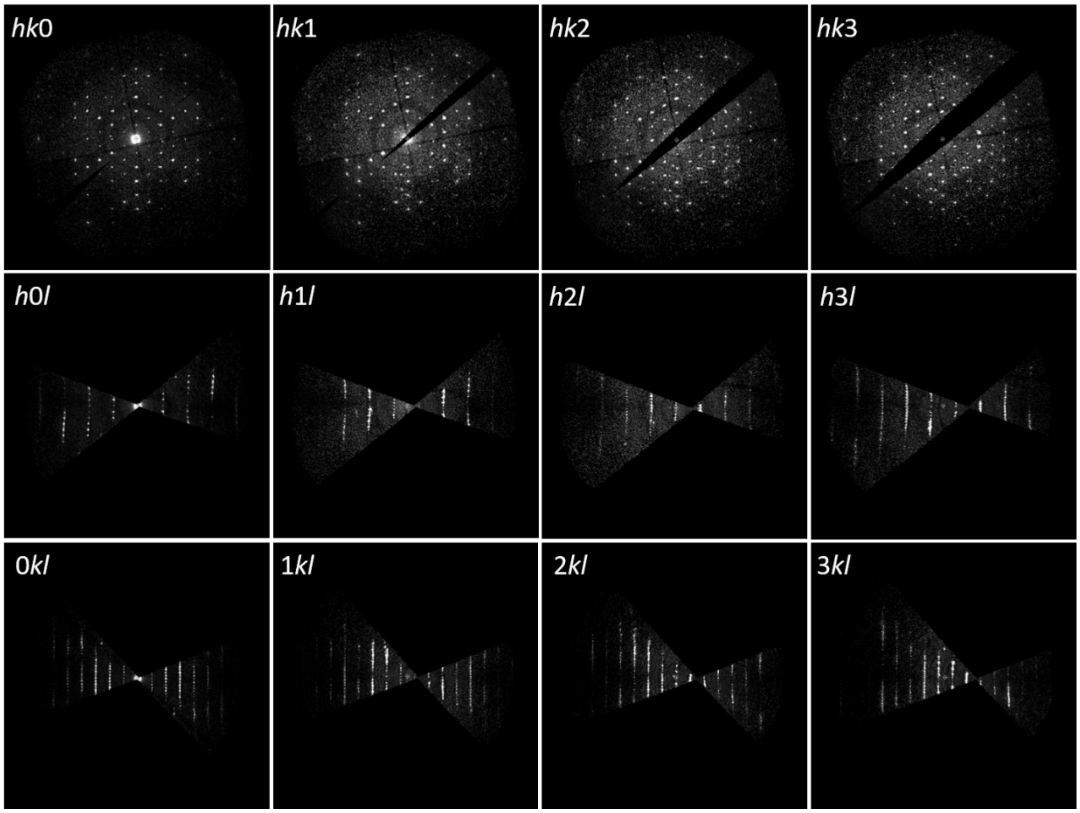

Figure S7. Planar cuts of the 3D ED reconstructions from the OctA sample.

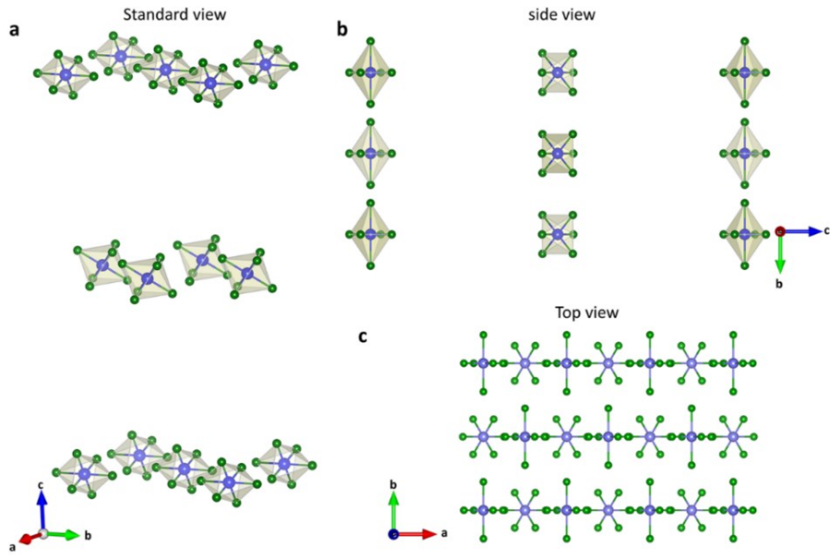

Figure S8. **a-c,** Crystallographic views of the inorganic layers in the OctA sample extracted from Vesta software.[9] Sn atoms are in violet and Br atoms are in green.

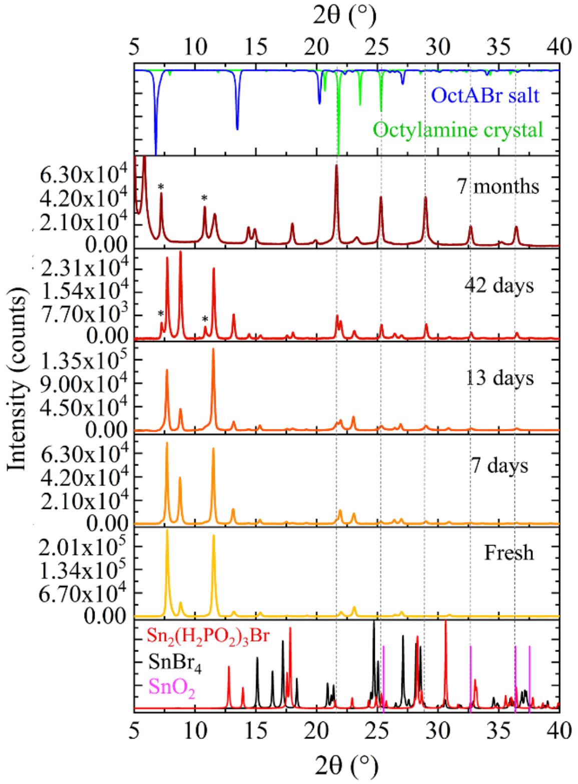

Figure S9. PXRD patterns collected at different times from as-synthesized OctA samples stored under ambient air conditions. References for different potential byproducts are also displayed. The vertical dotted lines highlight the position of new PXRD peaks observed from aged samples that match with SnO_2 (ICSD 98-018-1109), octylamine crystals (COD 7214425), and other Sn-based complexes like $Sn_2(H_2PO_2)_3Br$ 1D structures[8] formed as a result of the degradation process after 42 days under ambient air.

Figure S9 displays the PXRD patterns collected from as-synthesized OctA samples after 7, 13, and 42 days, and 7 months, to track potential ambient air-induced degradation of the structures reflected on the PLQY values. To note that the samples are not emitting under UV illumination after 7 months. From the collected PXRD patterns, we observe that the (00l) periodic peaks (at 8.80°, 13.17°, 17.58°, 21.97°, 26.47° and 30.94°) along with reflections from other crystallographic planes (at 15.35°, 19.23°, 23.10°, and 26.99°, and 26.99°), and the PXRD peaks originated from the stacking of platelets (7.65° and 11.57°) are preserved up to 42 days, with a decrease in their intensity from 13 to 42 days. These reflections disappear after 7 months. After 42 days, other PXRD peaks appear at low 2theta angle (7.25° and 10.83°, marked with (*)) along with reflections at higher angle (14.44°, 18.05°, 21.68°, 25.35°, 29.07°, 32.72°, and 36.40°), which increase significantly in intensity in the pattern collected after 7 months, with the appearance of a new peak at 5.76°.

Table S5. Organic cations investigated in this work for the fabrication of Sn-based layered perovskite-related structures using the protocol described in the main text. The amine structures are from Ref. [10].

Number	Amine name	Amine structure
1	Pentylamine	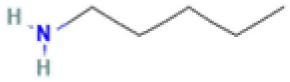
2	Undecylamine	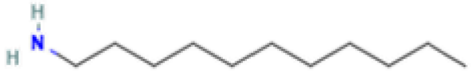
3	4-methylbenzylamine	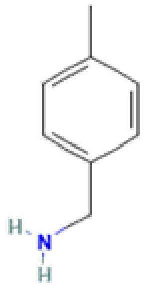
4	1-naphthylmethylamine	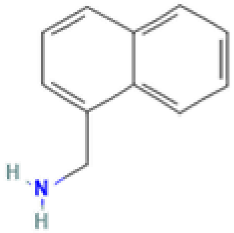
5	Cysteamine 2-	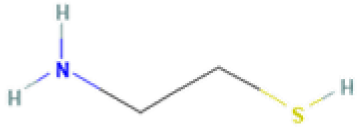
6	methylthiothylamine	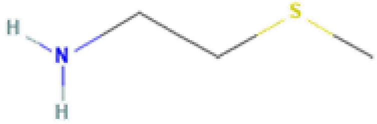
7	2-thiophenemethylamine	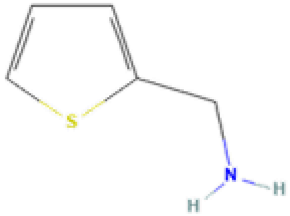

8	2-thiopheneethylamine	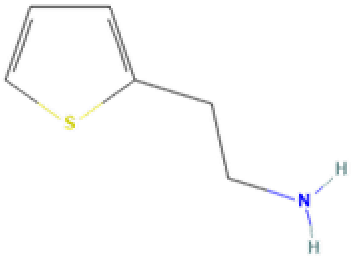
9	2-methoxyethylamine	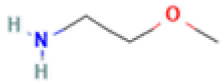
10	4-Aminobutanol	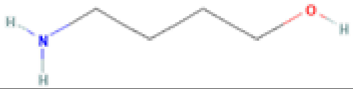
**11**	**2-phenoxyethylamine**	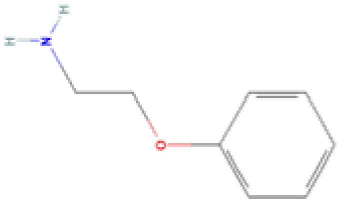
**12**	**4-Fluorobenzylamine**	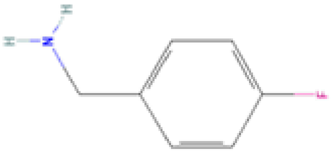

Table S6. Observations recorded from the synthesis performed by replacing octylamine by organic molecules from the amine family listed in Table S5.

**Amine number from Table S4**	**Observations**	**Photograph under nor-mal (right) and UV light (left)**
1	After 1 hour, a small amount of precipitates was observed that did not emit.	
2	Immediately after amine addition, white powders were formed, which emitted yellow under UV light.	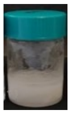 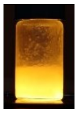
3	Sticky white/translucent product formed almost immediately and showing no emission under UV light.	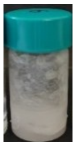
4	Immediately after the addition of amine, a pale-yellow product was formed that emitted pale or-ange under UV that correspond with the emission color of the amine bromide salt.	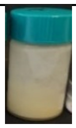 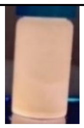
5	No product formed.	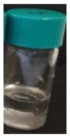
6	Small amount of white powder formed after 1 hour with no emission under UV.	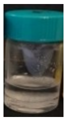 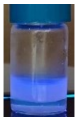
7	Small amount of white flaky powder formed after 1 hour with no emission under UV.	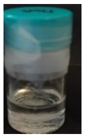
8	Formed yellow flaky and shiny product non-emitting under UV.	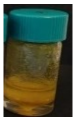 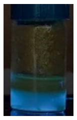
9	Very small amount of non-emitting powder locat-ed at the bottom of the vial.	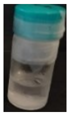 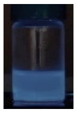

10	No product has formed	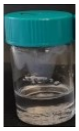
**11**	White needle like flakes grew slowly after the addition of amine. Emitting pink under UV	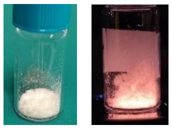
**12**	White flakes formed slowly after the addition of amine emitting pale greenish blue under UV	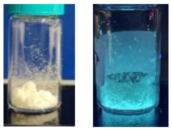

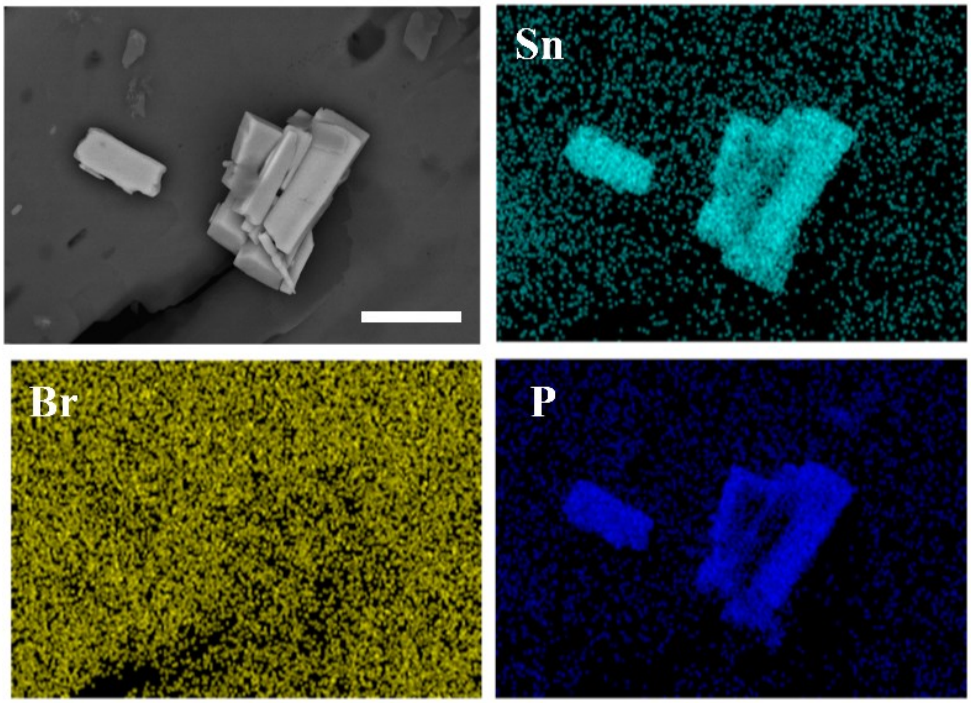

Figure S10. (top-left) SEM image of a representative area showing a group of POEA platelets and the corresponding EDS-SEM elemental mapping performed for Sn, Br and P. Scale bar: 25 µm.

Table S7. Elemental mapping analysis for Sn, Br, and P obtained via EDS-SEM performed on the POEA samples. According to this quantification, the resulting Sn:Br ratio in atomic % is 1:5.90.

Map Sum Spectrum	Line Type	Wt%	Wt% Sigma	Atomic %
Sn	L series	18.00	0.30	10.94
Br	K series	71.50	0.37	64.59
P	K series	10.50	0.23	24.47
Total		100.00		100.00

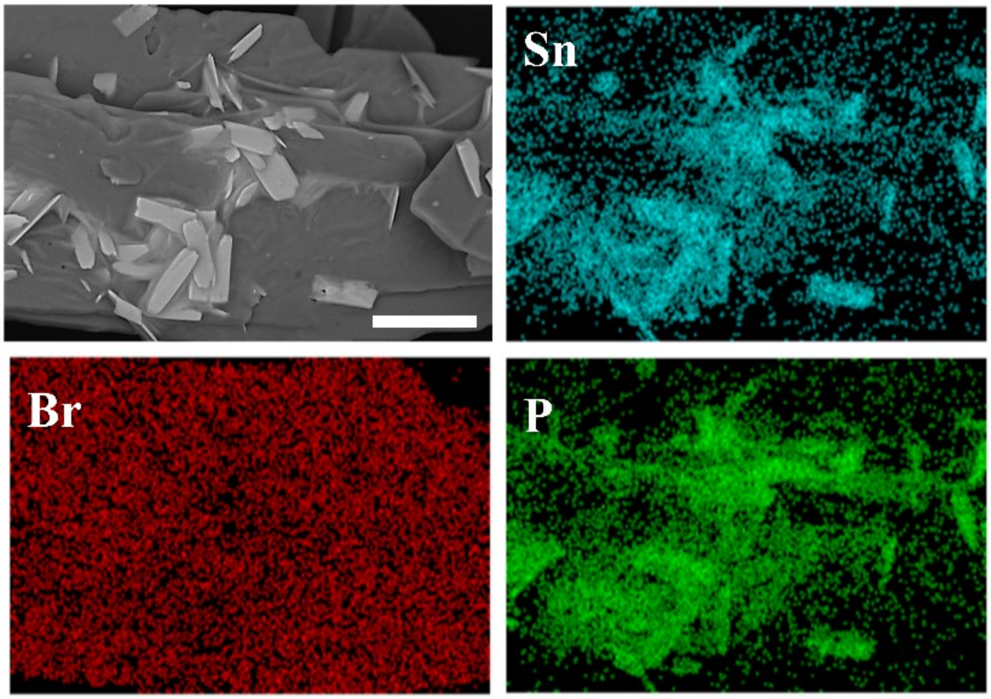

Figure S11. (top-left) SEM image of a representative area showing a group of FBA platelets and the corresponding EDS-SEM elemental mapping performed for Sn, Br and P. Scale bar: 50 µm.

Table S8. Elemental mapping analysis for Sn, Br, and O obtained via EDS-SEM performed on the FBA samples. According to this quantification, the resulting Sn:Br ratio in atomic % is 1:4.27.

Map Sum Spectrum	Line Type	Wt%	Wt% Sigma	Atomic %
Sn	L series	21.17	0.32	11.74
Br	K series	60.89	0.42	50.14
P	K series	17.94	0.27	38.12
Total		100.00		100.00

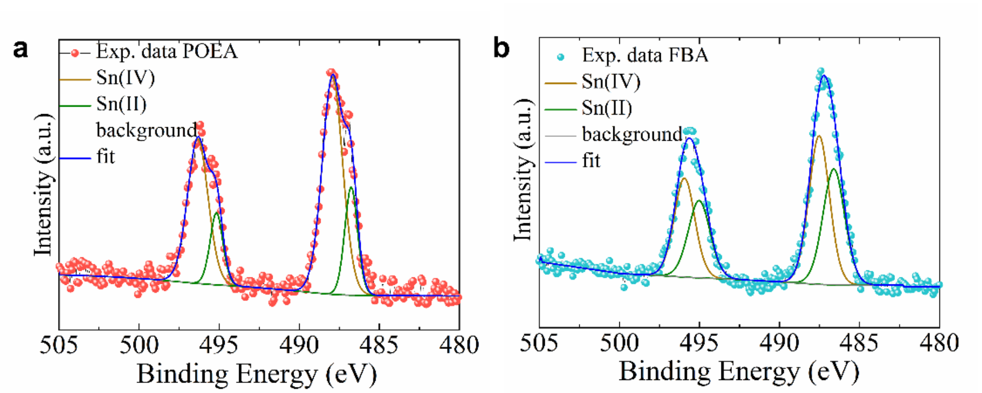

Figure S12. XPS spectra of Sn 3d state recorded on the POEA (a) and FBA (b) samples, evidencing the presence of Sn in the +2 and +4 state.

Table S9. Binding energy peak positions for Sn^{2+} and Sn^{4+} observed in the XPS spectra collected from OctA, POEA and FBA samples.

Sample		Sn^{2+}	Sn^{4+}
OctA	Sn $3d_{5/2}$	486.2±0.2 eV	487.6±0.2 eV
	Sn $3d_{3/2}$	494.6±0.2 eV	496.1±0.2 eV
POEA	Sn $3d_{5/2}$	486.8±0.2 eV	487.9±0.2 eV
	Sn $3d_{3/2}$	495.1±0.2 eV	496.3±0.2 eV
FBA	Sn $3d_{5/2}$	486.8±0.2 eV	487.5±0.2 eV
	Sn $3d_{3/2}$	495.0±0.2 eV	496.0±0.2 eV

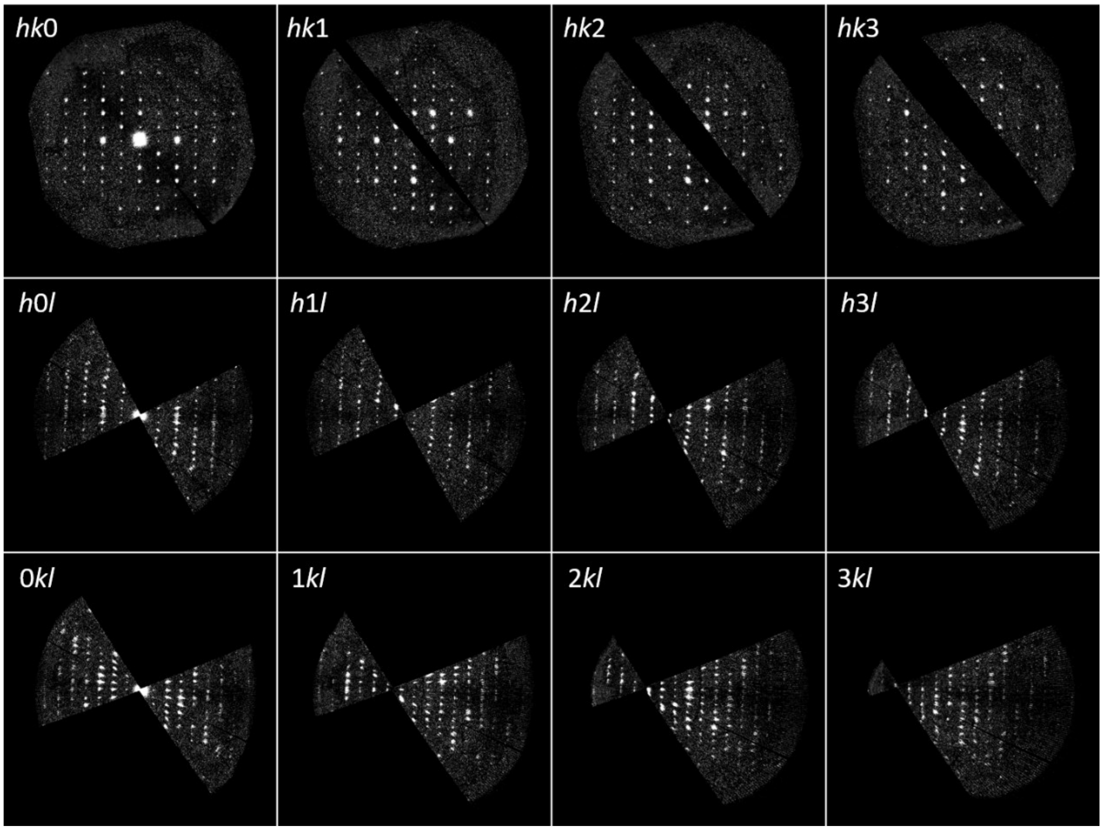

Figure S13. Planar cuts of the 3D ED reconstructions from the POEA sample.

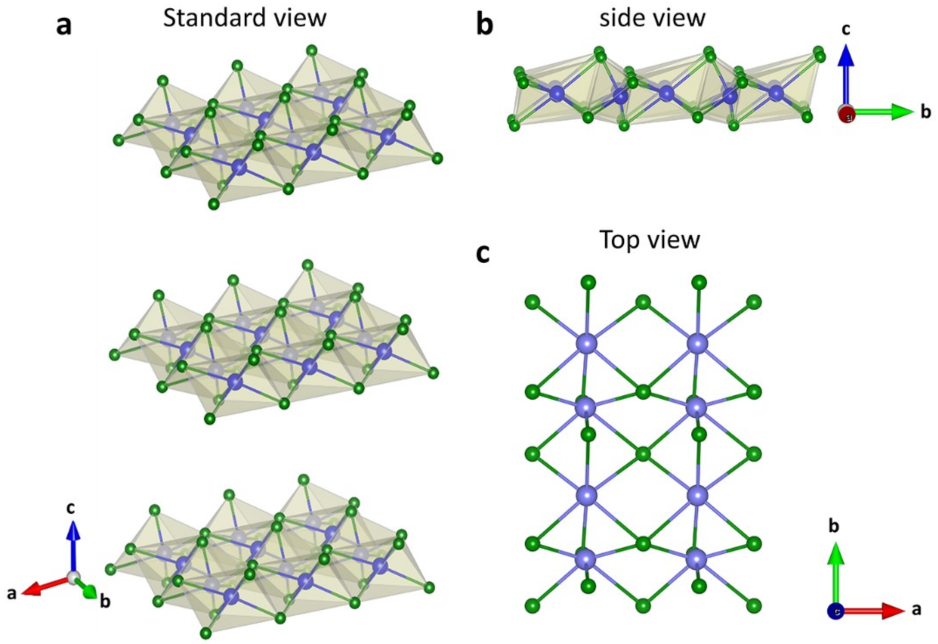

Figure S14. **a-c,** Crystallographic views of the inorganic layer in the POEA sample extracted from Vesta software.[9] Sn atoms are in violet and Br atoms are in green.

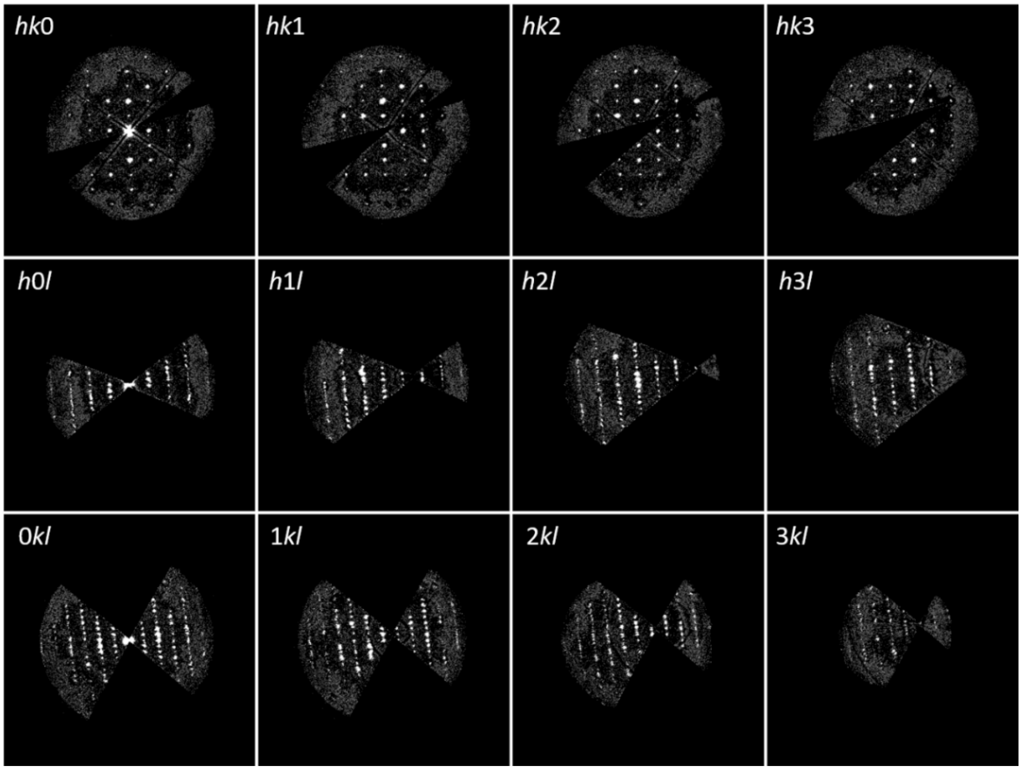

Figure S15. Planar cuts of the 3D ED reconstructions from the FBA sample.

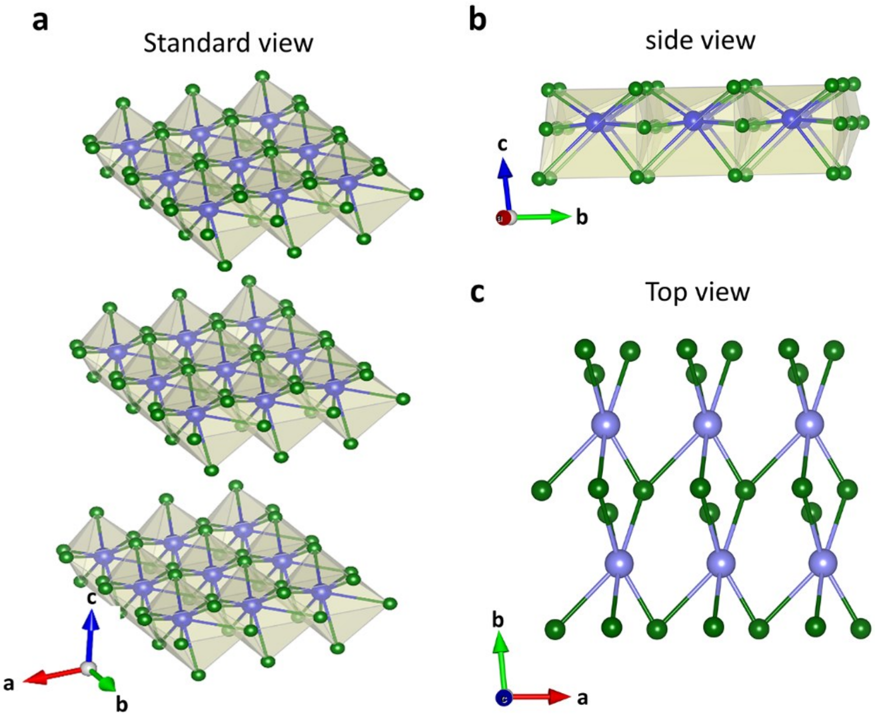

Figure S16. a-c, Crystallographic views of the inorganic layer in the FBA sample extracted from Vesta software.[9] Sn atoms are in violet and Br atoms are in green.

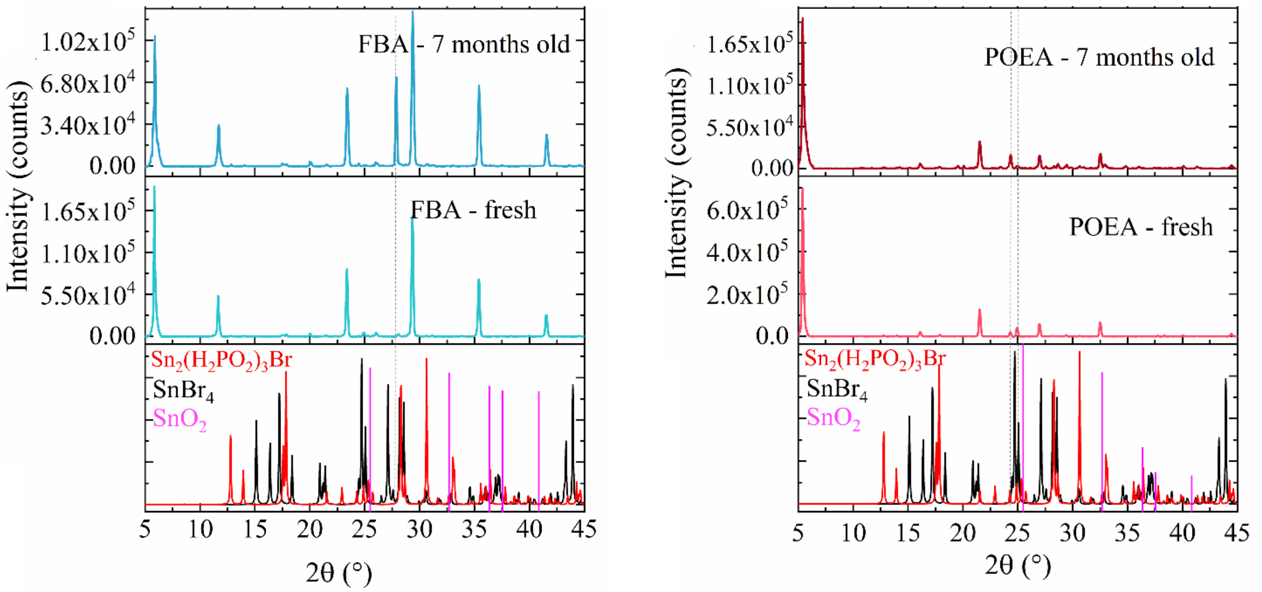

Figure S17. PXRD patterns collected from fresh and 7 months aged FBA (left) and POEA (right) organic cations. The samples were stored in glass vials under ambient air. References for different potential byproducts are also displayed. The vertical dotted lines highlight the position of new PXRD peaks observed from aged samples that match with $SnBr_4$ (ICSD-98-002-6033) in both samples and formed as a result of the degradation process after 7 months under ambient air. Also, the POEA sample shows a few reflections (around 32° 2theta) that indicate the presence of SnO_2 (ICSD 98-018-1109).

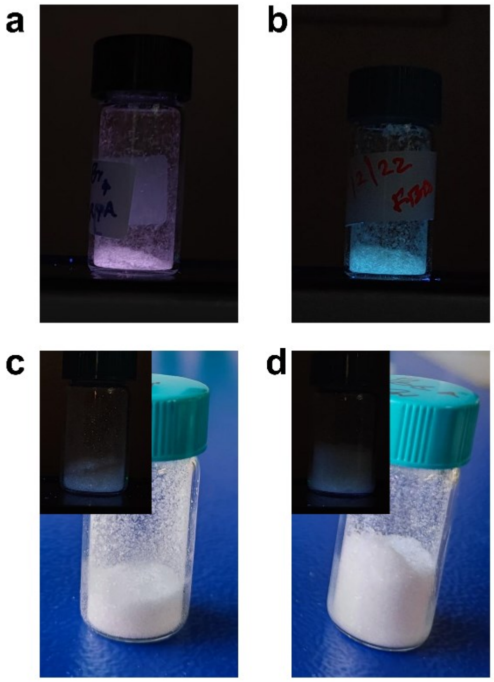

Figure S18. **a-b,** Photographs of the POEA (a) and FBA (b) crystals under UV light after 7 months stored in ambient air. **c-d,** Photographs of the corresponding bromide salts with POEA (c) and FBA (d) under normal light. The insets on each panel show the photograph under UV light, evidencing that the corresponding salts are not emitting in the same range.

Further details on the 3D ED analysis

3D ED data sets covered ranges from 51° to 107°. Camera length of 230 mm were used, allowing for a resolution in real space of about 1 Å. Continuous data collection was performed while the TEM goniometer was rotating with a constant angular speed of about 1.9°s^{-1}.[11] Simultaneously, the ASI MEDIPIX detector was sequentially acquiring ED patterns with an acquisition time 0.5 s, corresponding to angular integration of 0.95° per frame. ED data were energy-filtered by an in-column omega filter.[12] The tilt range of each data collection depended on the drift of the sample during rotation, which could never be completely compensated for. When necessary, more tilt sequences were collected on the same crystal at different tilt ranges and combined to extend the overall sampling coverage. In stepwise mode, the sample was rotated in fixed steps of 1°. After each tilt, a diffraction pattern was acquired, and the crystal position was tracked by defocused STEM imaging. This acquisition protocol implies a longer total exposure time per data collection but gives the possibility to track the position of the target crystal after each rotation step. Stepwise acquisition is therefore particularly suitable for small crystals. In order to proper integrate every reflection over the excitation error, during the experiment, the beam was precessed around the optical axis by an angle of 1°.[13] Precession was done using a Nanomegas Digistar P1000 device.

3D ED data were analysed using the *PETS2.0* software package.[14] Structure determinations of inorganic layers were achieved by standard direct methods (SDM), as implemented in the *SIR2014* software package.[15] The resolution limit was set at 1.1 Å. Data were treated with a fully kinematical approximation, assuming that I_{hkl} was proportional to $|F_{hkl}|^2$.

References

1. Liu, Y.; Wang, A.; Wu, J.; Wang, C.; Li, Z.; Hu, G.; Sui, S.; She, J.-X.; Meng, W.; Li, W.; Deng, Z., Alkylamine screening and zinc doping of highly luminescent 2D tin-halide perovskites for LED lighting. *Mater. Adv.* **2021,** *2* (4), 1320-1327.
2. Wang, A.; Guo, Y.; Zhou, Z.; Niu, X.; Wang, Y.; Muhammad, F.; Li, H.; Zhang, T.; Wang, J.; Nie, S.; Deng, Z., Aqueous acid-based synthesis of lead-free tin halide perovskites with near-unity photoluminescence quantum efficiency. *Chem. Sci.* **2019,** *10* (17), 4573-4579.
3. Hou, L.; Zhu, Y.; Zhu, J.; Li, C., Tuning Optical Properties of Lead-Free 2D Tin-Based Perovskites with Carbon Chain Spacers. *J. Phys. Chem. C* **2019,** *123* (51), 31279-31285.
4. Zhang, X.; Wang, C.; Zhang, Y.; Zhang, X.; Wang, S.; Lu, M.; Cui, H.; Kershaw, S. V.; Yu, W. W.; Rogach, A. L., Bright Orange Electroluminescence from Lead-Free Two-Dimensional Perovskites. *ACS Energy Lett.* **2019,** *4* (1), 242-248.
5. Xu, L.-J.; Lin, H.; Lee, S.; Zhou, C.; Worku, M.; Chaaban, M.; He, Q.; Plaviak, A.; Lin, X.; Chen, B.; Du, M.-H.; Ma, B., 0D and 2D: The Cases of Phenylethylammonium Tin Bromide Hybrids. *Chem. Mater.* **2020,** *32* (11), 4692-4698.
6. Nawale, V. V.; Sheikh, T.; Nag, A., Dual Excitonic Emission in Hybrid 2D Layered Tin Iodide Perovskites. *J. Phys. Chem. C* **2020,** *124* (38), 21129-21136.
7. Li, Z.; Deng, Z.; Johnston, A.; Luo, J.; Chen, H.; Dong, Y.; Sabatini, R.; Sargent, E. H., Precursor Tailoring Enables Alkylammonium Tin Halide Perovskite Phosphors for Solid-State Lighting. *Adv. Funct. Mater.* **2022,** *32* (18), 2111346.
8. Xie, J.-L.; Zhou, Y.-H.; Li, L.-H.; Zhang, J.-H.; Song, J.-L., A new method for the preparation of a $[Sn_2(H_2PO_2)_3]Br$ SHG-active polar crystal via surfactant-induced strategy. *Dalton Trans.* **2017,** *46* (29), 9339-9343.
9. Momma, K.; Izumi, F., VESTA 3 for three-dimensional visualization of crystal, volumetric and morphology data. *J. Appl. Crystallogr.* **2011,** *44* (6), 1272-1276.
10. Kim, S.; Chen, J.; Cheng, T.; Gindulyte, A.; He, J.; He, S.; Li, Q.; Shoemaker, B. A.; Thiessen, P. A.; Yu, B.; Zaslavsky, L.; Zhang, J.; Bolton, E. E., PubChem in 2021: new data content and improved web interfaces. *Nucleic Acids Res.* **2020,** *49* (D1), D1388-D1395.
11. Gemmi, M.; La Placa, M. G. I.; Galanis, A. S.; Rauch, E. F.; Nicolopoulos, S., Fast electron diffraction tomography. *J. Appl. Crystallogr.* **2015,** *48* (3), 718-727.
12. Lanza, A.; Margheritis, E.; Mugnaioli, E.; Cappello, V.; Garau, G.; Gemmi, M., Nanobeam precession-assisted 3D electron diffraction reveals a new polymorph of hen egg-white lysozyme. *IUCrJ* **2019,** *6* (Pt 2), 178-188.
13. Mugnaioli, E.; Gorelik, T.; Kolb, U., “Ab initio” structure solution from electron diffraction data obtained by a combination of automated diffraction tomography and precession technique. *Ultramicroscopy* **2009,** *109* (6), 758-765.
14. Palatinus, L.; Brazda, P.; Jelinek, M.; Hrda, J.; Steciuk, G.; Klementova, M., Specifics of the data processing of precession electron diffraction tomography data and their implementation in the program PETS2.0. *Acta Crystallogr. B* **2019,** *75* (4), 512-522.
15. Burla, M. C.; Caliandro, R.; Carrozzini, B.; Cascarano, G. L.; Cuocci, C.; Giacovazzo, C.; Mallamo, M.; Mazzone, A.; Polidori, G., Crystal structure determination and refinement via SIR2014. *J. Appl. Crystallogr.* **2015,** *48* (1), 306-309.
